# Biases in cultural transmission of information about a minimal ingroup

**DOI:** 10.1038/s41598-026-35241-x

**Published:** 2026-01-09

**Authors:** Mateusz Woźniak, Mathieu Charbonneau, Guenther Knoblich

**Affiliations:** 1 Institute of Applied Psychology, Jagiellonian University, Cracow, Poland; 2 Social Mind and Body Group, Department of Cognitive Science, Central European University, Vienna, Austria; 3 Social Cognition in Human Robot Interaction Group, Italian Institute of Technology, Genoa, Italy; 4 Africa Institute for Research in Economics and Social Sciences, Université Mohammed VI Polytechnique, Rabat, Morocco

**Keywords:** Self, Social identity, Cultural evolution, Minimal group, Transmission chain, Human behaviour, Psychology, Evolution, Cultural evolution

## Abstract

Group membership and our beliefs about the groups we belong to are the building blocks of our social and cultural identity. Here, we investigated whether transmission of information about how often different personality traits occur in a minimal ingroup and outgroup results in distinct patterns of cultural evolution. Participants transmitted information about the occurrence of positive, negative and neutral traits to other participants in linear transmission chains. First, we found a general tendency for occurrence of all traits to decrease across generations. However, our control experiment revealed that this general decrease was not specific to transmission of information about traits but represented a low-level response bias. Critically, this decrease across generations was smaller when participants were transmitting information about ingroup than outgroup traits, but only for positive and neutral traits. No significant difference emerged for negative traits. Together, these results show that minimal group membership can selectively bias transmission of information about ingroup and outgroup. We propose that this results from two processes: an ingroup-positivity bias and higher accuracy when transmitting ingroup-related information. Overall, our study provides an example of how examining mechanisms of cultural transmission can elucidate our understanding of processes of formation and evolution of social-cultural identity.

**Supplementary Information:**

The online version contains supplementary material available at 10.1038/s41598-026-35241-x.

## Introduction

Humans have a natural tendency to form groups and use these groups as anchors of their social identity^1–6^. However, social identity has a two-faced nature. First, it exists as mental representations of groups that we belong to and identify with^7–9^. From this perspective, social identity is a psychological phenomenon, something that is present in our minds. However, social identity is also a socio-cultural construct, as identities exist across the minds of people and are manifested through both material and immaterial representations^10–13^. This is especially salient for large social identities such as nations, religions and subcultures, which often develop complex systems of beliefs and symbolic representations, as exemplified by laws, ceremonies, flags, anthems etc. It can also be observed in small groups which can develop their own cultural entities through inside jokes, souvenirs etc. Such cultural representations associated with a social identity can also involve beliefs about other groups, especially about competing groups. For example, national identities might include stereotypes and beliefs about other nations (as illustrated by South and North Koreans, where national identities in both countries are strongly represented through a contrast with each other^14,15^. Socio-cultural beliefs about one’s own group are usually more positive and diversified than beliefs about outgroups^16,17^, which can lead to biases in decision making^17,18^, stereotyping, discrimination^19–23^ and dehumanization of outgroup members^24^. Such processes can increase in magnitude with time, leading to group polarization^25,26^ and sometimes even to tragic consequences, as evidenced by the history of violent conflicts and terror attacks.

There are many historical accounts discussing the evolution of beliefs about ingroup and outgroups^11,13,27^, as well as rich literature on psychological processes at play when people process information about ingroup and outgroup^16,28,29^. However, what is less explored is how these psychological processes might influence the evolution of beliefs about ingroup and outgroup. The motivation of the current study is to combine these two lines of research and to provide a contribution to investigating processes of cultural evolution of social identity, while using a minimal, experimental approach to this research problem. Specifically, the goal is to investigate whether transmission of information about ingroup and outgroup is characterized by systematic biases and whether these biases can lead to different trajectories of cultural evolution of beliefs about both types of groups. To achieve this, we conducted a study in which we used the minimal group paradigm^16,30,31^ to form minimal social identities among participants and asked them to transmit to other participants of the experiment (through transmission chains) information about the occurrence of positive, neutral, and negative psychological traits within their minimal ingroup as well as within the minimal outgroup.

The minimal group paradigm allows studying group identity in a laboratory setting. In studies using this paradigm, participants are assigned into a group that is typically characterized by some arbitrary marker (a color, a label, etc.) to differentiate it from the outgroup^16,30,31^. Such simple assignment affects a large number of processes including evaluation (positive, negative) and leads to biases in cooperative behavior (for a review see:^16^. While these biases are smaller and more limited than biases observed for real groups^32,33^ minimal groups allow investigating intergroup relations without the confounding influence of the history of previous intergroup interactions.

We used the minimal group paradigm together with the procedure of linear transmission chains^34–37^. Transmission chains, an experimental method that allows investigating processes of cultural evolution in a controlled experimental setting, can be seen as a well-controlled version of the “broken telephone” game. Each participant receives a piece of information and is required to transmit this information to another participant. The next participant in the chain is then asked to do the same to another participants, and so on, until the information reaches the last participant. Within this framework, a single sequence of participants transmitting information is called a transmission chain, and each participant represents a “generation”. By manipulating information given to the first participant (the “seed”) and running such transmission chains multiple times, it is possible to detect biases in information transmission, as well as whether these biases accumulate and converge towards a certain specific forms, or “attractors”^38–42^.

In the current study, we aim to investigate how psychological biases in processing of information about ingroup and outgroup might accumulate over time and lead to observable differences in beliefs about each group. To do so, we told participants that they belong to one of two villages (Green or Blue) and asked them to transmit information about the percentage of occurrence (POs) of various positive, neutral and negative traits, both within their own village and within the strangers’ village. Participants were told that the recipient of the information could be either a person from their group or the other group to model an instance of public, rather than private, communication (similar to posting an anonymous but publicly available post on a social network). We focused on psychological traits to keep the traits representing each valence as uniform as possible. Specifically, we aimed to answer three preregistered research questions:

### Research Question 1: Does repeated transmission of trait information about one’s minimal ingroup lead to different trajectories of cultural evolution compared to repeated transmission of trait information about a minimal outgroup?

Previous research in the field of social psychology has shown that processing of information about an ingroup as well as an individual’s behaviors towards (even unfamiliar) members of their ingroup are characterized by a range of cognitive biases^16,30^. These biases shape this information and such distorted information can subsequently be transmitted to other people – for example, a negative attitude of parents towards a certain group can be transmitted to their children^43,44^. However, what is less clear is whether this information can be distorted when it is being transmitted to another person. In our study we made sure to minimize the influence of biases on perception (by showing them unambiguous percentage numbers) and leave space for their influence on information transmission (by making the medium through which one transmits this information further prone to distortions – an unmarked line on which they had to indicate that number). Using the minimal group paradigm together with a controlled procedure of information transmission allows us to provide an answer to this question, as it allows to study the process of transmission of information about these two groups while keeping their initial characteristics the same.

### Research Question 2: Do evolutionary trajectories differ for traits with different valences (negative, neutral, positive)?

If mere membership in a minimal group biases the transmission of information about ingroups, outgroups, or both, then what kind of biases does it introduce? Previous studies found that people tend to process information about their ingroup in a more positive light than information about the outgroup^45–48^. If similar biases influence not only information processing but also information transmission, then the prevalence of positive traits should become higher for ingroup than for outgroup across generations. The opposite pattern should emerge for negative traits and there should be no difference for neutral traits. On the other hand, if different types of transmission biases are at play, then they would lead to a different pattern of results for each trait valence.

### Research Question 3a: Are there specific trajectories for each of the traits used in the study?

Specific biases in transmitting information might emerge at the level of valences of traits, but it is also possible that different individual traits are differently affected by those biases. For example, if participants show a general tendency to increase the reported occurrence of positive traits across generations, it might be the case that this effect is mostly driven by specific, most distinctively positive individual traits. Therefore, we also investigated the behavior of transmission chains for each trait individually.

### Research Question 3b: Do these specific trajectories reflect a priori beliefs regarding the presence of each trait in the general population?

People transmitting information about the occurrence of specific traits in the minimal ingroup and outgroup might be biased by their prior beliefs about how common these traits are in a general population. If this hypothesis is correct, then transmission chains should show a tendency to converge towards those values (i.e., those values should act as cultural attractors). We hypothesize that occurrences in general population, rather than in one’s ingroup, might be a more relevant category of reference, because people typically belong to multiple groups, which can differ widely in their characteristics. On the other hand, beliefs about general trait occurrence provide a more universal point of reference.

## Methods

### Preregistration

The experiment has been preregistered under the following link: https://aspredicted.org/51C_MHN.

### Participants

The experiment was conducted online via the *Prolific* crowdsourcing platform in May and June 2022. The preregistered target sample size was 180 participants divided into 18 transmission chains, with 10 generations in each chain. This sample size was chosen to allow detection of medium-large sized within-subject effects of group association (ingroup versus outgroup), which was our main comparison of interest. The power analysis was conducted using G*Power 3.1 (calculated for the difference between two dependent means, two-tailed, alpha = 0.05, beta = 0.8, effect size dz = 0.75). The sample size was chosen before the start of data collection.

We also conducted a simulation-based power analysis in R using the *simr* package for the linear mixed-effects model. The model included fixed effects of Group (ingroup vs. outgroup), Valence (neutral, positive, negative; neutral as the reference level), Generation (0–10, numeric), and all interactions among these predictors, with random intercepts for Chain and Participant:

Y ~ Group × Valence × Generation + (1∣Chain) + (1∣Participant)

Residual error was drawn from a normal distribution with *SD* = 1, so that fixed-effect coefficients can be interpreted as standardized effect sizes. For the key three-way interaction of interest, how the group difference over generations changes specifically for *positive* versus *neutral* valence (we expected the effect for negative versus neutral to be similar), we set the coefficient for the term *Groupoutgroup: Valencepositive: Generation* to β=−0.15 (a small-to-moderate standardized effect). All other fixed effects were set to 0 for the power calculation. Using 1,000 Monte-Carlo simulations with α = 0.05 (two-tailed), the design achieved an estimated power of 92% (95% CI [0.90, 0.94]) to detect this three-way interaction. A power-curve analysis varying the number of participants indicated that approximately 150–160 participants would be sufficient to reach 80% power for an interaction of this magnitude.

206 participants completed the experimental procedure and yielded full data sets. The first preregistered exclusion criterion was that participants whose transmitted ratings deviate on average (across both groups and all traits) by more than 10% points would be excluded. This led to the exclusion of 6 participants. Second, participants who did the task twice were also excluded, and new participants were retested in their place, what applied to 17 participants (to speed up data collection data for six transmission chains was collected simultaneously and in *Prolific* it was not possible to automatically block participants who participated in one chain from signing up for another). Three participants were excluded due to errors in the experimental script that led to incomplete data. After accounting for the exclusions, the final sample size consisted of 180 participants.

Among the final sample 81 participants identified as female, 97 as male and 2 chose to not provide gender-related information. The mean age of all participants was 33.2 years (*SD* = 12.2, min = 19, *Q1* = 24, *Median* = 29.5, *Q3* = 41, max = 76). Participants needed on average 8.5 min to complete the whole experiment. Participants represented 27 nationalities, with the six most common being UK (46), Poland (25), Portugal (15), South Africa (13), Italy (12) and USA (11). 78 participants were full-time workers, 28 part-time workers, 15 unemployed and 8 not in paid work (e.g., retired or disabled). 41 participants were students.

All participants gave informed consent after reading a general information about the study and before starting the experiment. The study was approved by the Ethical Research Committee of Central European University (approval no. 2020/08), in accordance with the standards from the Declaration of Helsinki.

Prestudy and both control experiments were conducted with different samples than the main experiment. Description of the tested samples for these experiments can be found online (Supplementary Material 1, Supplementary Material 2, and Supplementary Material 3).

### Design

The design of the study was a 2 (Group: Ingroup vs. Outgroup) x 3 (Trait valence: Positive vs. Neutral vs. Negative) x 11 (seed information + Generations 1 to 10). The experiment had a two-level random effects structure with participants nested within transmission chains. Group and Trait Valence were within-participants variables, while Generation was a within-chain variable.

The main dependent variable were participants’ reports about the occurrence of a trait in a particular group. It was measured as percentage of occurrence (PO) of traits, separately for ingroup and outgroup, transmitted by each participant to the next generation.

The factor Generation was on an interval scale and could be treated either as a factor (treating it as a nominal variable) or as a covariate (treating it as a continuous variable). The preregistered analyses assumed treating Generation as a factor rather than a covariate because of the possibility that the trajectories of transmission chains might be highly non-linear. However, our results showed that the average trajectories were largely linear. Therefore, in the main text we report the results treating Generation as a covariate. The results of all analyses, also involving analyses treating Generation as a factor, are available online (Supplementary Material 4, Supplementary Figs. S7–S10, Supplementary Tables S6–S9).

In this study, we report all measures, manipulations and exclusions. All methods were in accordance with the standards from the Declaration of Helsinki.

### Procedure

#### Experimental task

**Fig. 1:**
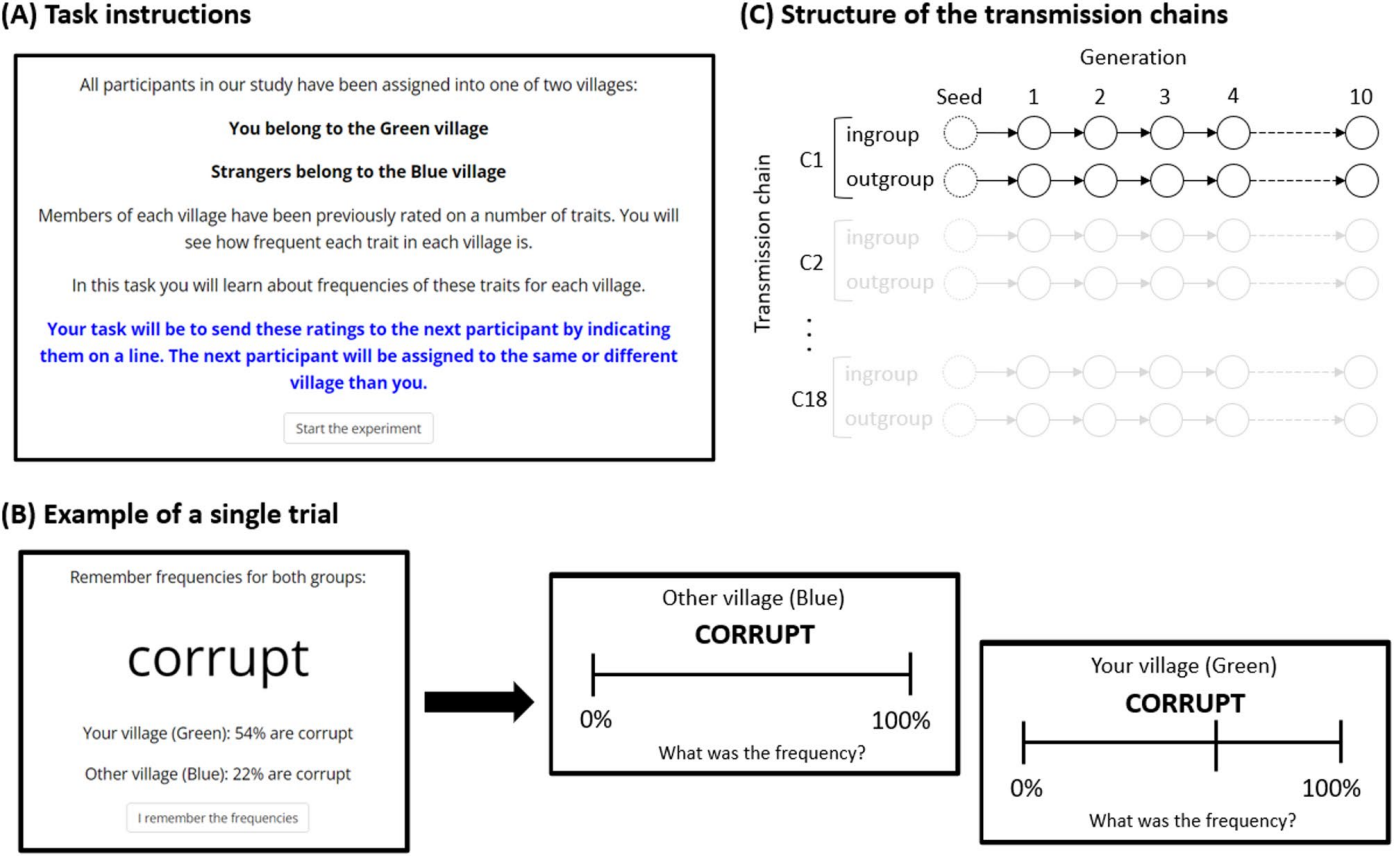
The procedure of the experiment.

At the beginning of the experiment participants were presented with task instructions, which are displayed in Fig. 1A. The task instructions first told participants to memorize which of two villages is “their” village, and which is a village of “strangers” (Green and Blue village – the assignment of colors was counterbalanced across transmission chains). In this way the minimal groups were established. Afterwards, they read that each group had been previously rated on a number of traits and that they would see how frequent each trait was in each group. They were then told that their task would be to memorize these frequencies and then send them to the next participant, who can be either from their or a different village.

Afterwards, they proceeded to the task, which consisted of 21 trials, reflecting 21 traits that were used in the study. Each trial started with a screen presenting the name of the trait, together with the percentage of its occurrence (PO) for each village (Fig. 1B). Displayed POs were natural percentage numbers between 0% and 100%. After participants read and memorized the percentages, they pressed a button. This led to the presentation of two subsequent screens in which participants task was to indicate the frequency of that trait for their own and the strangers’ village (displayed in random order) on a continuous line representing the POs. The beginning and end of the line were labeled as 0% and 100%. Clicking on the line left a mark. Participants could revise their response as many times as they wished. They submitted their response by pressing a button below the line.

#### The procedure of transmission chains

The experiment involved 18 transmission chains with 10 generations each (i.e., 10 episodes of information transmission, see Fig. 1C). Seed values for transmission chains were generated before the experiment using the following rules: (1) the average PO of the traits of each valence (negative, neutral, positive) had to be 50%, (2) the average standard deviation of POs for each valence had to be approximately 10, (3) the POs within each valence could not repeat. This also enforced requirement (4) that the PO for any given trait could not be the same for ingroup and outgroup. Seed values created using these rules were then assigned to traits. The assignment of seed values to traits was counterbalanced across participants to ensure that the same combination of seed values was equally often used for ingroup and outgroup traits, and equally often used for negative, neutral and positive traits. The same rules were applied to seed values of the three additional traits (religious, political, attractive).

The first participant in each transmission chain was shown the seed values. For each subsequent generation the responses from the previous participant were rounded to a natural number and used as the POs to be learned and transmitted. Participants were not explicitly told whether the POs that they transmitted originated from a previous participant.

#### Pre-study: selection of traits for the experiment

To select the traits used in the experiment, we conducted a pre-study on Prolific in which 26 participants evaluated 72 traits on a scale from 1 (“very negative”) to 7 (“very positive”). These traits were initially classified by the experimenters as positive, neutral, negative (20 from each category), or ambivalent (12 traits). The full list of traits together with descriptive statistics of the ratings are available online (Supplementary Material 1, Supplementary Table S1). Based on those participants’ responses, six traits for each valence were chosen. Six negative traits from among the traits with the lowest ratings were selected, and analogously the six positive traits from those with the highest ratings. Neutral traits were chosen among those rating close to the middle point (4 points). An additional criterion was to avoid using traits that are opposites of one another (e.g., dishonest/honest). We also included three additional traits in the study, which were not used to calculate averages for each valence but were of potential theoretical interest: attractive (a positive, but non-psychological trait), religious and political (ambivalent traits, reflected in the highest variability of how they were judged). Table 1 presents the full list of selected traits, together with their average ratings.

**Table 1:** Average ratings of the 21 traits used in the experiment. Each trait was rated on a scale from 1 (very negative) to 7 (very positive).

POSITIVE		NEUTRAL		NEGATIVE		ADDITIONAL	
Friendly	6.31	Trendy	4.35	Corrupt	1.39	Religious	3.54
Intelligent	6.42	Busy	4.04	Dishonest	1.46	Political	3.62
Honorable	6.27	Traditional	4.35	Lazy	2.04	Attractive	5.54
Skilful	6.31	Predictable	4.23	Without empathy	1.96		
Charismatic	6.0	Introverted	3.81	Impolite	1.62		
Creative	6.15	Mystical	4.19	Cowardly	2.08		
	6.24		4.16	Mean:	1.76		

#### Control Experiment 1 (CE1): beliefs about the frequency of occurrence of traits in general population

Preregistered research question 3b asked whether the traits in the transmission chain would converge towards values that are believed to be characteristic of the general population. This required estimating what these values were. In order to do this, CE1 was conducted via Prolific. 51 participants were asked to indicate how frequent each of the 21 traits used in the transmission chain are thought to be in the general population. They responded by indicating the value on a line similar to the one used for the transmission chains. Full results are presented online (Supplementary Material 2, Supplementary Fig. S1, Supplementary Tables S2 and S3).

#### Control Experiment 2 (CE2): investigating the response bias in a number-to-position task

The second control experiment tested whether participants are biased when responding on the linear scale used in the main task. The CE2 consisted of 99 trials in which participants saw a number between 1% and 99% (randomly selected without replacement) and had to mark this number on the same line as the one used for the transmission chains. Again, only the extremes were labeled and the selection mark appeared only after participants made a response with a mouse click. It was possible to correct one’s response as many times as needed and there was no time limit to respond. The trial ended when participants clicked on a “Continue” button. Each trial started with a fixation cross presented for 500ms. 30 people completed the CE2. Full results are presented online (Supplementary Material 3, Supplementary Figs. S2–S6, Supplementary Table S4, S5).

### Apparatus and stimuli

All experiments were programmed in JavaScript using the jsPsych toolbox^49^. The response scale (continuous line) was implemented using the VAS plugin to jsPsych^50^. The experimental script was hosted on Cognition.run (transmission chains experiment, control experiment 1) and Pavlovia (control experiments 2 and 3) hosting platforms.

### Data analysis

All data and data analysis scripts are available under the following link: https://osf.io/azpcy/.

## Results

### Confirmatory analyses

#### Research Questions 1 and 2

To answer the first two questions in the preregistration we proposed a classic ANOVA approach, and a Linear Mixed Model (LMM) approach. We first report the results of the preregistered ANOVA analysis. However, this analysis treats generation as a categorical variable and therefore does not consider strong interdependence of data from neighboring generations (being conceptually similar to analyzing within-subject data using between-subject analysis methods), whereas LMM allows us to take such interdependencies into account. Second, in the LMM analysis we modelled generation as a covariate, rather than a factor, because the data revealed that the effect of generation was linear, and not non-linear – contrary to our expectations.

#### Preregistered analysis 2 using ANOVA

A preregistered 3-way ANOVA with factors Valence (positive vs. neutral, vs. negative), Group (ingroup vs. outgroup), and Generation (0 to 10) for the PO values revealed a significant main effect of generation (*F*(10, 187) = 39.8, *p* < .001, partial *η*^*2*^ = 0.68), a significant main effect of group (*F*(1, 187) = 9.23, *p* = .003, partial *η*^*2*^ = 0.05), a significant main effect of valence (*F*(1.72, 321.8) = 5.28, *p* = .008 Greenhouse-Geisser corrected, partial *η*^*2*^ = 0.03), and a significant interaction between group and valence (*F*(1.78, 332.5) = 9.2, *p* < .001 Greenhouse-Geisser corrected, partial *η*^*2*^ = 0.05). The remaining effects were not significant: the interaction between group and generation (*F*(10,187) = 1.19, *p* = .30, partial *η*^*2*^ = 0.06), the interaction of valence and generation (*F*(17.2, 321.8) = 0.2, *p* = 1 Greenhouse-Geisser corrected, partial *η*^*2*^ = 0.01), and the 3-way interaction (*F*(17.8, 332.5) = 0.4, *p* = .99, partial *η*^*2*^ = 0.02). The main effect of generation reflected a generalized reduction in PO values across generations, and the main effect of groups showed overall significantly lower PO values for outgroup than ingroup.

**Fig. 2:**
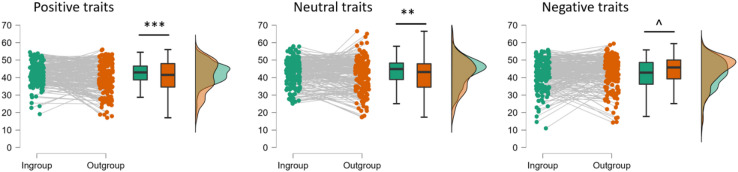
Percentage of occurrence of traits (PO) across all generations in ingroup and outgroup, separately for positive, neutral, and negative traits.*** *p* < .001, ** *p* < .01, ^ *p* < .1.

To further investigate the interaction between group and valence we conducted post hoc tests comparing POs for ingroup vs. ingroup for each of the three trait valences. The results revealed that the difference between groups was significant for positive (*t* = 3.29, *p* < .001, all Bonferroni corrected, Cohen’s d = 0.32), and neutral traits (*t* = 3.21, *p* = .006, Cohen’s d = 0.32) indicating higher PO values for ingroup. For the negative traits POs were lower for ingroup than outgroup, but the difference was only marginally significant (*t* = 2.55, *p* = .054, Cohen’s d = 0.25). Figure 2 illustrates the results of the analysis.

#### Preregistered analysis 2 using linear mixed models (and treating generation as a covariate)

We conducted a linear mixed effects model analysis with factors Group (Ingroup vs. Outgroup) and Valence (Positive vs. Neutral vs. Negative), Generation treated as a covariate and Participants and Chains treated as random factors. Because the full model did not converge we report results of the model with only chains as random factors. We allowed the chains to have both random intercepts and random slopes (as a function of generation). The analyses were run in R (v4.3.0) using the lme4 package (v.1.1–33).

The results of the model (AIC = 7726.83, BIC = 7808.11, Pseudo-R² (fixed effects) = 0.35, Table 2) revealed a significant main effect of generation reflecting a systematic decline of values across generations. An interaction between group and valence was significant when contrasted against the negative valence and was marginally significant when contrasted against the positive valence. However, these two-way interactions were qualified by a three-way interaction between Group, Valence and Generation, which was significant for negative valence as a reference category, and marginally significant for positive valence as a reference category. The remaining effects were not significant.

**Table 2:**
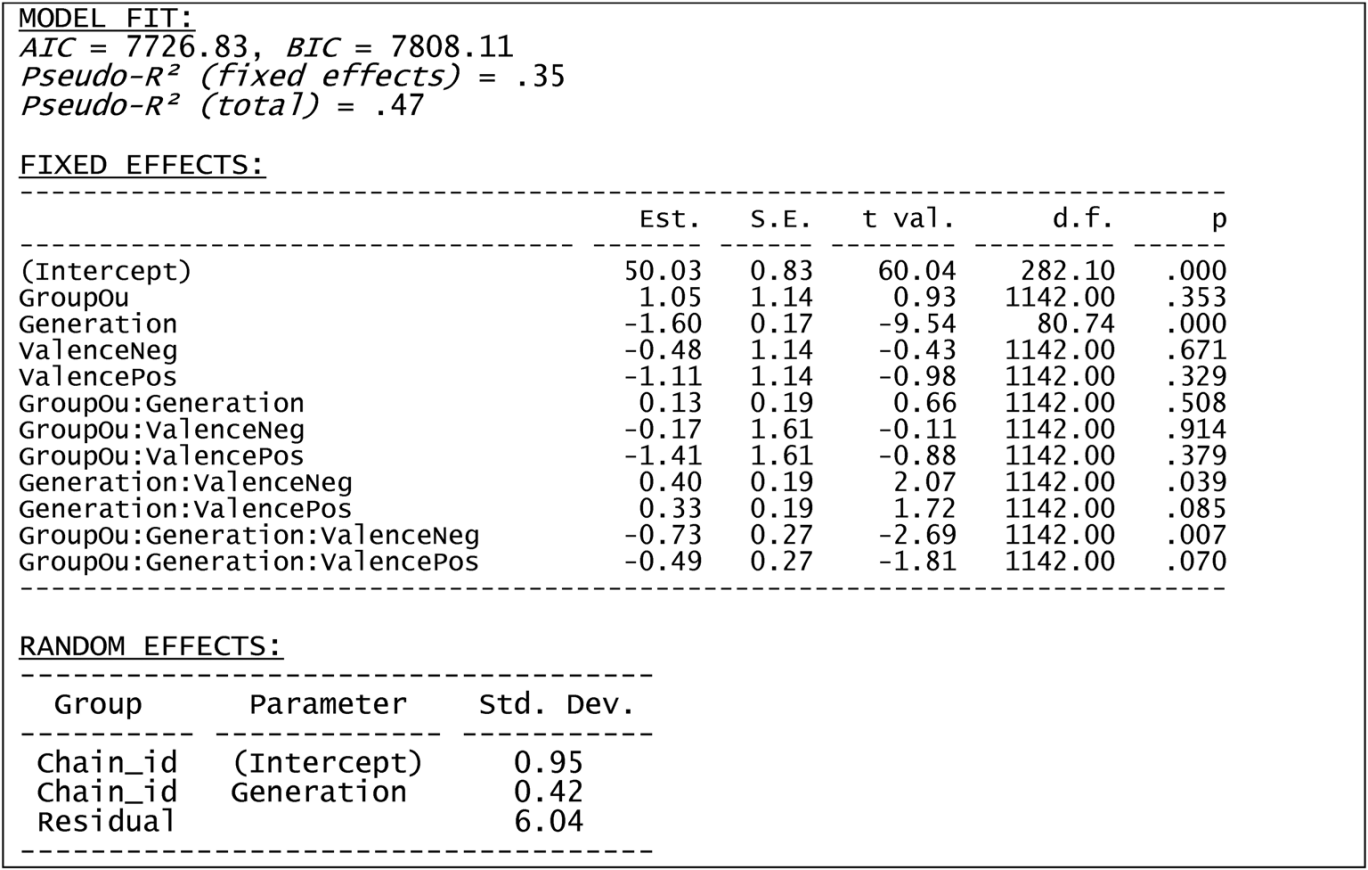
The results of the full linear mixed model. All p-values calculated with Satterthwaite method.

**Fig. 3:**
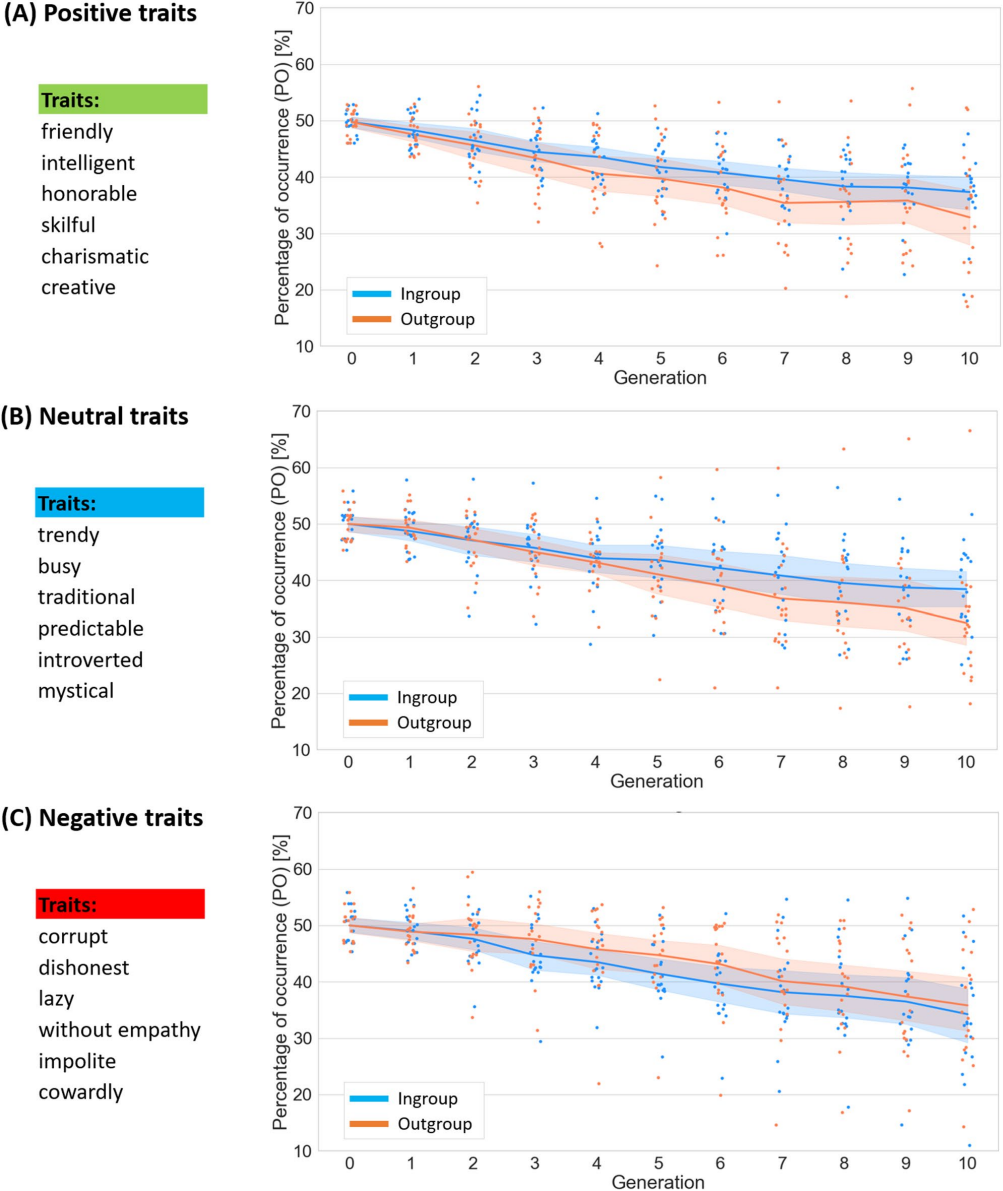
Percentage of trait occurrence (POT) averaged for each valence as a function of generation.

To further explain the three-way interaction we conducted additional analyses separately for data of each valence (see Fig. 3 for illustration of the results). The motivation of these follow-up analyses was to determine whether the effect of group on transmission is different for different valences. This is the main research questions that we aimed to address and which drove the design of our study. Moreover, we justify these analyses by the presence of significant and marginally significant 3-way interaction effects in the main analysis. For each of the follow-up analyses we used a linear mixed model with Group as a factor, Generation as a covariate and chains as a random effect with random slope and random intercept (the inclusion of participants as a random effect was not justified as indicated by a Chi2 test, *p* = 1). For positive traits the model (AIC = 2413.43, BIC = 2445.28, Pseudo-R² (fixed effects) = 0.39) revealed a significant main effect of generation (*t*(32.4)=-8.66, *p* < .001) and a significant interaction between group and generation (*t*(360)=-2.46, *p* = .01). The main effect of group was not significant (*t*(360) = 0.41, *p* = .684). The significant interaction reflected stronger negative slope for outgroup than for ingroup. The same pattern of results was present for neutral traits (AIC = 2496.65, BIC = 2528.50, Pseudo-R² (fixed effects) = 0.35): a significant main effect of generation (*t*(32.33)=-7.41, *p* < .001) and a significant interaction between group and generation (*t*(360)=-3.67, *p* < .001), with non-significant main effect of group (*t*(360) = 0.91, *p* = .365). For negative traits (AIC = 2537.63, BIC = 2569.48, Pseudo-R² (fixed effects) = 0.33) the main effect of generation was significant (*t*(29.22)=-8.59, *p* < .001), while the main effect of group (*t*(360) = 1.03, *p* = .305) and the interaction (*t*(360) = 0.73, *p* = .464) were not.

#### Research Question 3a

Specific trajectories of transmission chains for each trait used in the study, as well as the detailed statistical results, can be found online (Supplementary Material 5, or in an interactive dashboard under the following address: https://mmwozniak-cultevoself.streamlit.app/). In order to investigate the results at the level of individual trials we conducted a linear mixed effects analysis with group as a factor, generation as a covariate and transmission chain as random effect (the reported results are with random intercepts, but the pattern of results regarding the interaction was the same when random slopes were included). Because the dashboard was programmed in Python, we used the statsmodels (version 0.12.2) mixedLM package for linear mixed models. This analysis revealed that the interaction between group and generation was significant or marginally significant for the following positive traits: friendly (*p* < .001), intelligent (*p* = .007), skillful (*p* = .001), creative (*p* = .054); negative traits: corrupt (*p* = .041), dishonest (*p* = .079), cowardly (*p* = .008); neutral traits: busy (*p* < .001), predictable (*p* < .001). From among the additional three traits the interaction was significant for attractiveness (a non-psychological positive trait: *p* < .001) and marginally significant for religious (a neutral, but highly divisive trait: *p* = .077). For the majority of traits significant interaction reflected that percentage of occurrence was characterized by slower decrease in ingroup than in outgroup, with the exceptions being all negative traits and a single positive trait: skillful.

#### Research Question 3b

The final preregistered analysis involved investigating whether believed frequencies of occurrence of traits in a general population act as cultural attractors in our task. Our preregistered analysis involved comparing the average percentages of occurrence in generation 10 with values obtained in Control Experiment 1 in which we asked 51 participants to estimate what is the percentage of people that possess each trait in a general population. Table 3 presents the descriptive statistics from the control experiment (results of statistical analyses can be found online (Supplementary Material 2)). The preregistered t-tests revealed that for almost all traits estimated occurrence was significantly higher than the value at generation 10 (one exception in which they were significantly lower was: “mystical”, and for “without empathy” and “corrupt” the difference was not significant). Because all traits showed the trend to decrease across generations this pattern cannot be explained by not having enough generations to converge to an attractor. Instead, it shows that the percentage of occurrence of almost all traits continued to decreased even after reaching these values. The second way to test our prediction that the believed percentage of occurrence of traits in a general population will act as an attractor was to investigate whether there is a correlation between these values and the values at generation 10. This correlation was not significant in our data (*r* = .16, *p* = .49).

**Table 3:** Average ratings of occurrence of the 21 traits used in the experiment (“EO” columns: results of the control experiment 1) and average frequency of occurrence in generation 10 (“Gen10” columns: average for ingroup and outgroup).

POSITIVE	EO	Gen10	NEUTRAL	EO	Gen10	NEGATIVE	EO	Gen10
Friendly	64.06	34.9	Trendy	48.94	31.92	Corrupt	43.12	33.92
Intelligent	53.41	39.52	Busy	67.27	40.41	Dishonest	49.10	31.88
Honorable	48.75	30.13	Traditional	53.04	30.34	Lazy	52.55	32.77
Skilful	57.22	36.06	Predictable	65.04	41.18	Without empathy	39.45	42.32
Charismatic	46.45	32.83	Introverted	49.84	29.38	Impolite	44.25	30.05
Creative	52.75	37.11	Mystical	32.14	39.5	Cowardly	51.57	39.23
Mean:	53.77	35.09		52.71	35.46		46.67	35.03

### Exploratory analyses

#### Analysis of the trajectories of transmission chains

**Fig. 4:**
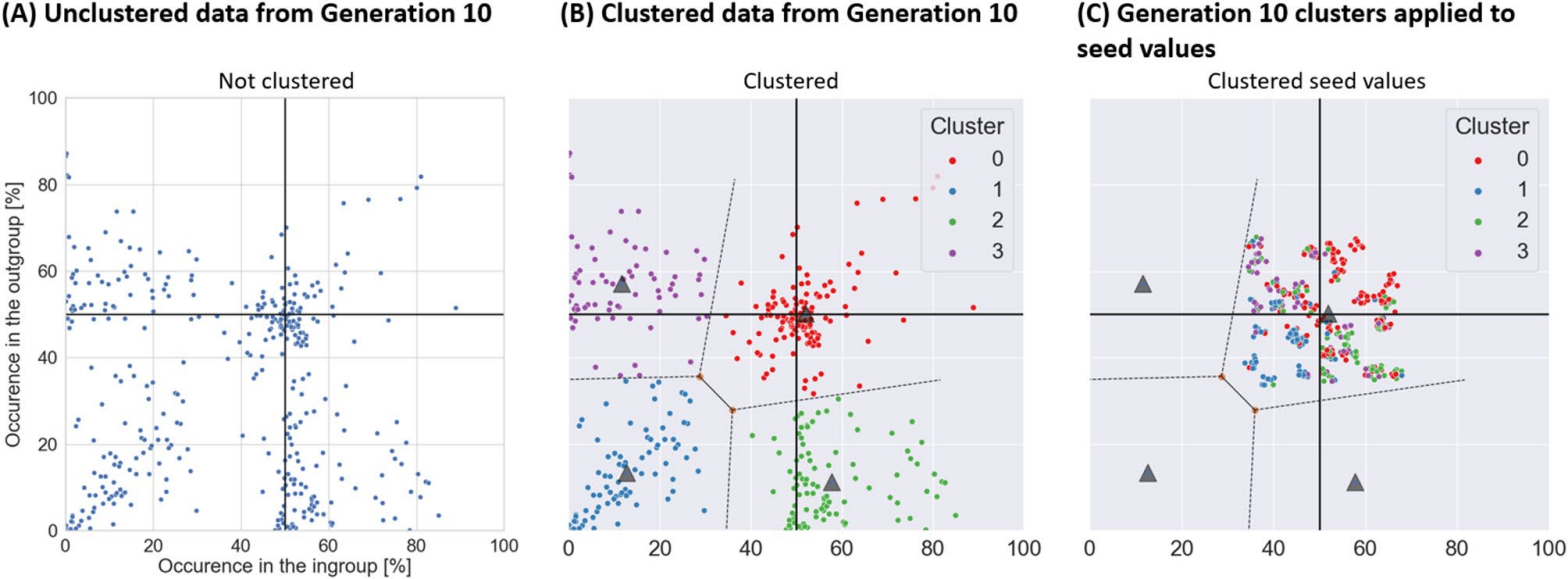
The results of the cluster analysis. (**A**) The percentage of occurrence of all traits at generation 10. (**B**) The results of k-means clustering of data from generation 10 revealed four clusters. (**C**) The distribution of seed values (jittered) coloured by the cluster to which each point will belong at generation 10 shows strong relation between the starting position and the position at generation 10.

According to the cultural attraction theory^38–41^ cultural content might converge towards specific points, or “cultural attractors”. Cultural attractors can be observed when cultural evolution repeatedly steers content towards one or more specific forms. In order to investigate whether cultural attractors emerged in our study we conducted k-means cluster analysis on the data from the final generation of all transmission chains and all traits. The maximal calculated silhouette score (= 0.599) was for k = 4, so we extracted 4 clusters (Fig. 4). The four clusters’ centres were points (x: ingroup occurrence, y: outgroup occurrence): [51.9, 50.2], [57.8, 11.4], [11.6, 57.1], and [12.6, 13.5] indicating existence of four attractors in our data.

At the next step, we tested whether the initial percentage of trait occurrence in ingroup and outgroup at seed predicted the cluster in which a given transmission chain ended at generation 10 (Fig. 4C). A decision tree model trained on full data set (max depth = 2, criterion = Gini) led to 60.6% accuracy, significantly higher than the chance level of 25% (more details and additional analyses, including the cross-validation analyses can be found online (Supplementary Material 5)). These results show that seed position had a strong influence on the subsequent trajectory of a transmission chain. Seed values that started at or above 50% tended to converge towards 50%, while values that were below 50% tended to decrease towards zero across generations.

**Fig. 5:**
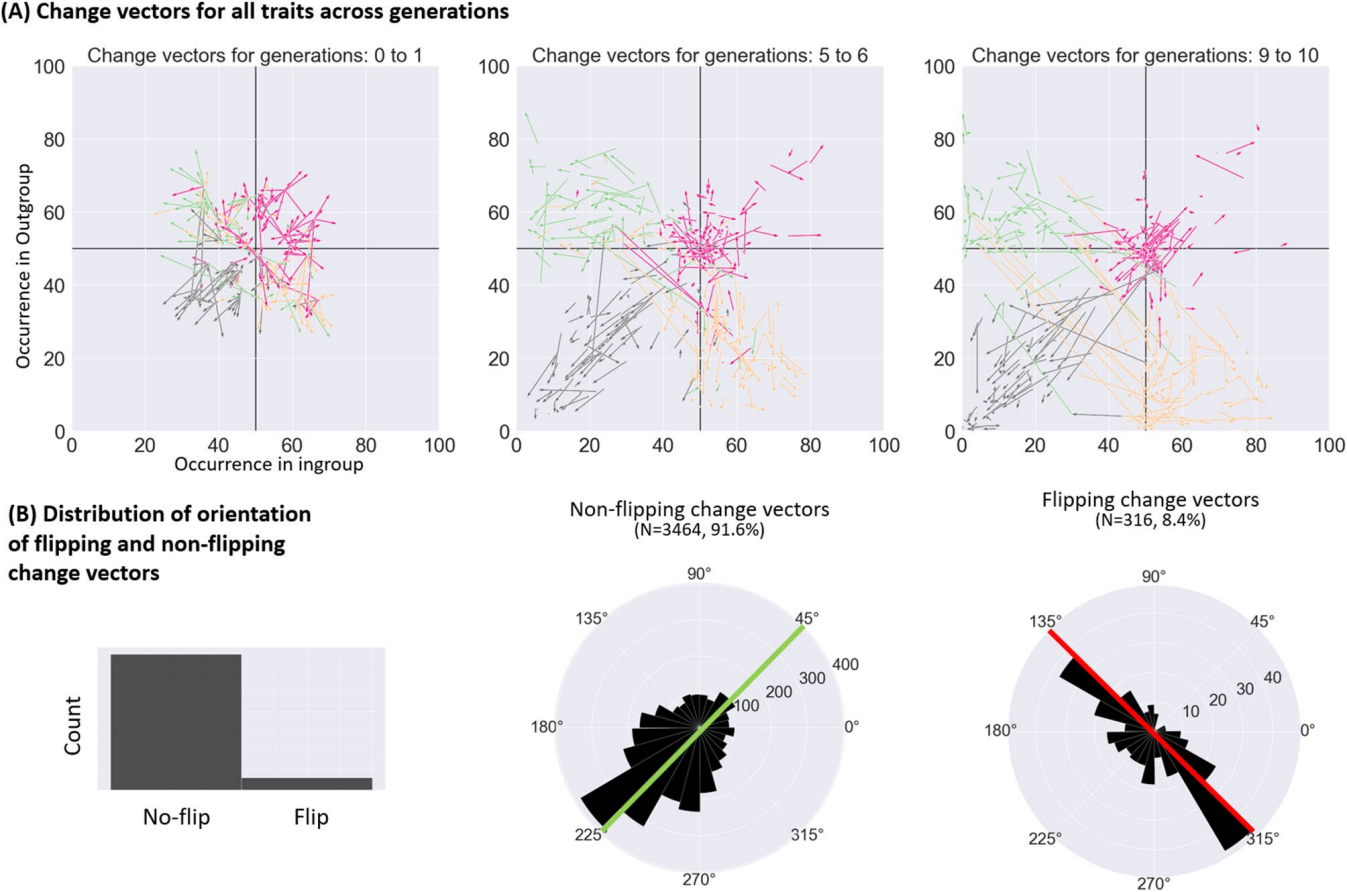
(**A**) Illustration of change vectors at the start of transmission chains (seed to Generation 1), in the middle (Generation 5 to 6) and the end (Generation 9 to 10). (**B**) Distribution of change vectors that led to a “flip” between the groups (i.e., when a participant misremembered which group had higher and which group had lower PO), and the change vectors that did not. “Non-flipping” changes overwhelmingly led to the decrease of POs for both groups, as indicated by the data clustered in the lower left quarter. “Flipping” changes were much less common and led to a radically different distribution of change vectors. Most of them were at the red diagonal line, which shows situation in which the PO in one group increases to the same extent as it decreases in the other group. This situation suggests that in these cases participants changed POs for ingroup and outgroup, either through memory lapse or doing it intentionally.

The two-dimensional plane of ingroup and outgroup trait occurrences (like the one shown in Fig. 4) can be treated as a landscape of attraction. In order to further explore its properties, we computed vectors representing each instance of transmission (Fig. 5). Each vector represents a change from values presented to a participant (vector’s tail) into values reported by that participant (vector’s tip). Plotting these vectors (Fig. 5A) from one generation to the next one (for all traits) illustrated the findings from cluster analysis, showing that at each generation values above 50% tended to decrease towards 50%, while values below 50% tended to decrease towards 0%. This behaviour represents the main effect of generation from the analysis perform to answer research questions 1 and 2.

Closer inspection of the data reveals that while the tendency of percentages to decrease towards 50% and 0% was a predominant effect, there was also a second pattern of changes: instances of jumps traversing the diagonal line from [0,0] to [100,100]. These jumps will be called “flips”, because they led to the flip in which group’s percentage is higher and which is lower. Figure 5B presents histograms of directions of all vectors of transmission, separately for cases in which the values flipped (8.4% of the cases) and in which they did not (91.6%). While the flipping events were much less common, they demonstrated a radically different distribution of orientations. The non-flipping events usually led to the decrease of percentages for both groups. On the other side, the flipping events predominantly resulted in situations in which the same value was added to one group and subtracted from the other group. This kind of effect is likely the result of a memory distortion, in which a participant correctly memorized the percentages, but misremembered which percentage referred to which group.

#### Control experiment 2

**Fig. 6:**
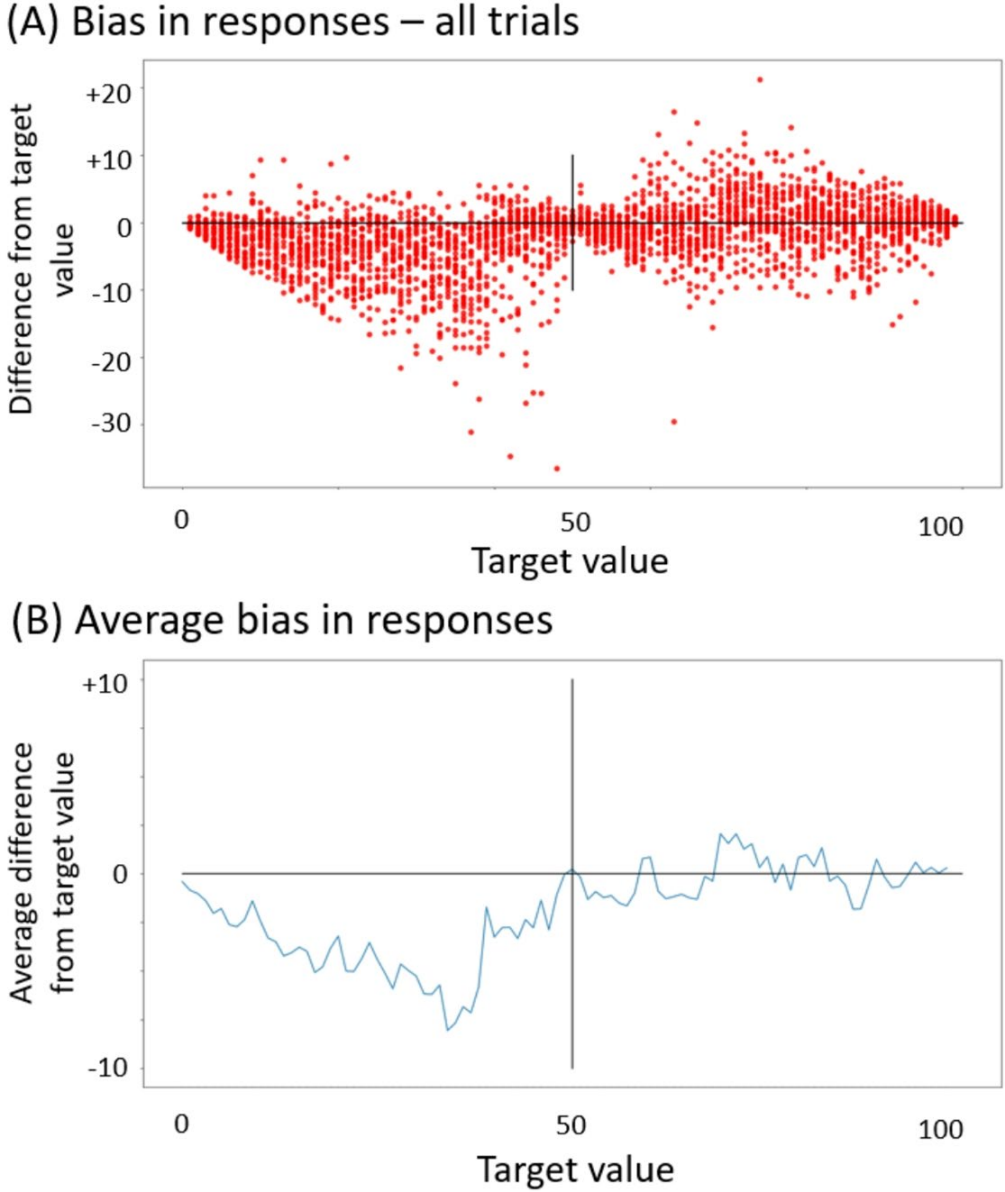
The results of the control experiment 2 (CE2). (**A**) All individual data points. The x-axis corresponds to the target number, and y-axis to the difference of the response from the target number (**B**) The average difference of the response from the target number.

In order to investigate whether the systematic decrease of values (the main effect of generation) was specific to transmitting information about groups, or whether it was a more general bias, we ran CE2 in which 30 participants from the Prolific platform were presented with random numbers between 1% and 99% and asked to indicate them on a line (the size and positioning of the line were identical to the transmission chains experiment). The results showed that participants were accurate when reporting numbers between 50% and 100% but had a systematic tendency to underrepresent numbers smaller than 50% (Fig. 6). This result shows that this effect is not specific to transmission of information about traits but reflects instead a low-level response bias.

## Discussion

We conducted a preregistered transmission chain experiment to investigate whether cultural transmission of information about characteristics of minimal ingroup and outgroup differ and what role does valence of this information play in this process. Specifically, we modeled a situation of public information transmission, when the group membership of the receiver is unknown. Our first two research questions were:

### Research Question 1

Does repeated transmission of trait information about one’s minimal ingroup lead to different trajectories of cultural evolution than repeated transmission of trait information about a minimal outgroup?

### Research Question 2

Does evolutionary trajectories differ for traits with different valences (negative, neutral, positive)?

Our results showed that these two factors interact with each other. While all types of traits for both groups showed a general tendency to decrease in percentage across generations (an effect that we will discuss in the next section), positive and neutral traits were characterized by slower decrease when they described an ingroup than an outgroup. At the same time, this difference was not significant for negative traits, and numerically it even went in the opposite direction, showing that lack of effect was not due to low statistical power.

How to interpret this pattern of results? Our data does not allow to determine a single uncontroversial explanation. Therefore, we propose several potential explanations of our findings. First, it might reflect higher accuracy when transmitting information about ingroup than outgroup. We will call this explanation an “ingroup-accuracy” effect. While POs of all types decreased across generations, reflecting a systematic distortion of the original information, this distortion was smaller for information about the ingroup. This might be a consequence of facilitated processing of ingroup-related information, as has been demonstrated in a wide range of earlier studies. Previous research on self-related processing found extensive evidence of the self-prioritization effect, i.e. faster reaction times and increased accuracy when processing information related to oneself^51–54^, as well as distinct brain encoding of such information^55–57^ and self-reference effect –better memory encoding of self- than other-related information^58–60^. Very similar effects have been found for processing of information about one’s ingroup, including minimal ingroup^61–63^ (this is expected as one’s group membership forms an important part of one’s self-representation:^2,6,64,65^. Moreover, there is evidence of facilitated perceptual processing of faces belonging to one’s ingroup^66,67^. These results suggest that information related to one’s ingroup might be processed with more attention to details than information related to the outgroup, leading to an overall better accuracy of transmission. However, when inspecting our results in more detail we observed that this was only true for neutral and positive traits, and not for negative traits. Thus, even if higher processing accuracy is responsible for our pattern of results, it cannot be the only explanation – otherwise negative traits would also exhibit the same effect.

What other factor can account for differences in patterns of results observed for different valences? Our data does not allow for a fully conclusive explanation, but there are some potential candidates. A significant interaction between group membership and valence points towards a second potential interpretation: that transmission of information about the ingroup might be characterized by the motivation to present one’s ingroup in more positive light, also known as the “ingroup-positivity” bias^16,29,30,61^. In its basic form this interpretation predicts that across generations information about the ingroup should become more positive and less negative than about the outgroup. It means that POs for positive traits should become higher, and for negative traits lower in ingroup as compared to the outgroup. This effect has been consistently reported in previous studies involving the minimal group paradigm. These include the effect of group membership on explicit attitudes^46,68,69^, implicit attitudes^45,70,71^, positive trait attributions and inferences^72–74^, and more (see:^16,30^ for a review). While this effect might have different motivations depending on the context^16,75,76^, in our case it might be associated with a tendency to avoid providing information that might be potentially harmful to one’s ingroup to the members of the outgroup – especially because our participants were informed that the recipient of their rating might belong to any of the two groups. In its basic form the ingroup positivity effect should be of equal size (but different direction) for positive and negative traits. However, there is literature showing that ingroup-positivity and outgroup-negativity might act differently depending on the context, and might be underpinned by slightly different mechanisms. While in many contexts people display both, in some contexts people display only ingroup-positivity, without the accompanying outgroup negativity. For example, some studies found ingroup favoritism when distributing positive outcomes, but did not show equivalent ingroup favoritism when distributing negative outcomes^77,78^. On the other hand, in some contexts the ingroup-bias might emerge only for the negative characteristics. For example^79^, found that people are more likely to attribute negative (but not positive) ingroup characteristics as being caused by human nature, hence humanizing ingroup flaws.

The ingroup-positivity bias provides a second potential explanation of our results, although it also does not fully fit our data, as it predicts no difference between groups for neutral traits, and our results showed that such difference was present. Hence, each of these two interpretations individually fails to account for our observed pattern of results. However, if these processes took place simultaneously then they could lead to the pattern that we observed. In such case, the first potential explanation is that the presence of ingroup positivity together with the higher accuracy effect when transmitting ingroup-related information could lead to cancelling out of these effects for the negative traits, just like we observed in our study. At the same time, in the presence of a general tendency for the values to decrease across generations, for neutral and positive traits these two effects would act in the same direction, leading to slower decrease of values for ingroup than for outgroup – the exact pattern that emerged in our study. However, if these effects add up in a straightforward, cumulative fashion then the difference between groups should be larger for positive than for neutral traits. This was not the case in our data. This might indicate that either this explanation is incorrect or that there are additional factors that play a role. First, the ingroup-positivity bias in our study might have been asymmetrical in relation to valence, and hence affected only the negative traits. As shown by the prospect theory^80–82^, people are more sensitive to losses than gains. As such, transmitting negative information about ingroup might have had a higher potential cost, motivating people to give biased responses in favor of their ingroup when relaying information about negative, but not positive traits. Indeed, as mentioned before, there is previous literature showing similar effects in certain contexts in minimal group paradigm^79^. In our study, one potential motivation for such effect might be the intention to avoid giving “ammunition” against my ingroup to recipients of my ratings that could come from the outgroup. There are at least two alternative explanations that are more methodological. First, there may be a ceiling effect to how much these two effects (for higher ingroup accuracy and positivity) can stack up and influence participants’ responses. A second explanation is that a trait that acted as an outlier in our data, the trait “skilled” which showed faster decrease for ingroup than outgroup, was responsible for this lack of a cumulative effect.

Overall, our results extend previous literature by showing that the transmission of information about groups introduces its own types of psychological ingroup biases. Our results and the design of the experiment allow us to only speculate about the exact nature of these biases, although without providing definitive proofs, as other competing explanations are also possible. Here, we propose that our data can be explained by simultaneous influence of two well-established effects: (1) higher accuracy when transmitting information about ingroup than outgroup and (2) ingroup-positivity, but limited to a situation when participants have to transmit information about negative characteristics of their ingroup. The simultaneous presence of two biases might be a consequence of telling participants that the transmitted information would reach either a person from their own group or from an outgroup. Invoking two types of addressees might have shaped the exact form of biases. For example, if the information were to reach an ingroup then it makes more sense to be more accurate, while for an outgroup it makes more sense to demonstrate a higher level of ingroup-positivity, either in symmetric or asymmetric form. Future research should test this hypothesis, as well as fully validate the proposed explanations of our results.

### Response bias

The discussed group differences were present within the context of a strong tendency for the values of all traits (irrespective of the valence and group assignment) to decrease across generations. This decrease was present in our main transmission chains experiment but also in the Control Experiment 2 (additional results can be found online (Supplementary Material 3)) where participants performed the same number-to-line estimation task outside of the context of valence, group membership or cultural transmission. Participants were simply presented with a percentage number and had to indicate where it fell on the same response line that has been used in the main experiment. CE2 revealed that participants accurately represented numbers falling between 50% and 100% but had a strong tendency to under-represent numbers between 0% and 50%. This tendency could be as strong as 6.5 points on average for numbers falling between 30% and 40%. Because this bias was present in a task that did not require manipulation of information about groups or traits, it must have been driven by low-level processes related to numerical cognition. Indeed, similar biases have been previously observed in adult population, although their magnitude was much smaller^83^. This difference in the magnitude of the bias might have been caused by the fact that we conducted the study online. It might have motivated the participants to perform the task faster and less accurately, hence exacerbating the response bias that would otherwise be much smaller. Interestingly, this bias was much stronger in our control experiment than in the transmission chain experiment. For example, in the main experiment in the range between 30% and 40%, participants undershot by on average 3 points – significantly less than the 6.5 points in CE2. This suggests that some characteristics of the transmission chain study might have reduced the magnitude of the bias. One possibility is that the context of the study, including transmitting information about one’s ingroup, motivated the participants to put more effort into the task, and subsequently to reproduce the numbers on the line more accurately.

Reporting response biases, such as the one from our study, is important for the field of cultural evolution, for both methodological and theoretical reasons. Methodologically, for researchers using transmission chains^37,84^ the presence of these biases illustrate the importance of controlling for low-level effects present during information transmission, as some observed patterns of cultural evolution may be mere artifacts of these effects, or alternatively may cancelled by such effects. On a more general level, they show the importance of understanding the specificity of the medium of cultural transmission^85–88^. In the case of our study, using a number-to-line estimation task performed on a home computer resulted in a very specific type of response bias that resulted in the tendency for the transmitted values to fall, either until they reach 50% or 0%. Using the terms from the theory of cultural evolution^38,41,89^ these two values acted as cultural attractors in our study. Attractors can be understood as abstract stabilization points towards which cultural content tends to converge in the course of cultural evolution. For instance, portrait painting with direct eye gaze (where the subject is “making eye-contact” with the view) have become predominant because of the attention grabbing and strong emotional responses they elicit^90^. Attractors can come as a result of various types of processes, termed “factors of attraction”^38^. In the previous case, cognitive factors such as our reactions to averted and directed gaze would have led portraiture traditions to evolve towards direct eye gaze. But these factors can also be ‘ecological’ (or non-cognitive), such as motoric constraints on the evolution of rhythms^91^. In our study, a simple response bias for underrepresenting numbers falling between 0% and 50% when indicating them on a line resulted in the emergence of four attractors illustrated in Fig. 4. If our task was used in a more applied scenario, it might have led to formation of a very specific pattern of beliefs about distribution of traits among groups: traits would be reported as either present in approximately 50% of population or almost absent – and this effect would be driven purely by the medium used for cultural transmission, i.e. the number-to-line task.

Interestingly, further exploratory analysis of distribution of participants’ responses revealed a second type of response bias: a situation in which participants “flipped” the percentages of occurrence of traits that were assigned to ingroup and outgroup. This was a consequence of the task design in which participants first had to memorize the occurrence of a given trait in both groups, and then had to reproduce these percentages (in random order) at the subsequent step. This made it possible for participants to either misremember which value was associated with which group, or for them to intentionally switch them. Such process indicates presence of a different type of attractor, which is driven by different cognitive factors of attraction (e.g., memory lapse or intentional intervention, rather than number-to-line response bias). As such, our study shows how specific design choices made in respect to how transmission of cultural information takes place, can lead to introduction of additional very specific types of cultural attractors and trajectories of cultural evolution that are independent of any biases related to the transmitted content per se (see also^37^, Sect. 4). It also raises the question to what extent the medium of cultural transmission can inadvertently shape its content.

### Cultural evolution of group information about specific traits

The last research question in our study was concerned with specific trajectories of cultural evolution of individual traits. Specifically, we asked two questions:

### Research Question 3a

Are there specific trajectories for the 21 specific traits used in the study?

When inspecting individual traits, our results generally confirm the finding that valence affected how group membership influenced the trajectories of cultural evolution but also showed that this effect was highly variable across different traits. These results are presented and discussed online (Supplementary Material 5). Interested readers can also explore these results in an interactive dashboard available at the following address: https://mmwozniak-cultevoself.streamlit.app/.

### Research Question 3b

Do they reflect a priori beliefs regarding the presence of each trait in the general population? (estimated in a control study)

Finally, our results did not provide support for our prediction that beliefs about the occurrence of traits in a general population act as cultural attractors. However, lack of this effect might be due to the fact that our results were very strongly affected by the number-to-line task response bias that led to a general trend for all values to decrease across generations. It remains possible that in the absence of such bias the populational averages might act as attractors. More research is needed to provide a definitive answer to this question.

### Future directions

Our results contribute to the understanding of group phenomena that are grounded in cultural processes. However, more research is needed to fully elucidate the strength and relative influence of mechanisms that allow group membership to influence cultural transmission. Our study involved several design choices that could be manipulated in future research. First, we used a number-to-line estimation task as a method of cultural transmission. It allowed us to introduce variability into the transmitted material, but it also introduced a strong systematic response bias towards estimating percentages between 1% and 50% as smaller than expected. Reproducing our experiment with a method of information transmission that is not affected by such systematic bias might determine the relative strength of the two factors that explain our results: higher accuracy of transmission of ingroup-related information and ingroup-positivity bias. Specifically, in the absence of a drift towards low or high values, these two explanations lead to contradictory predictions: higher-ingroup-accuracy predicts that for all valences percentages should stay closer to the original value for ingroup than outgroup, regardless of the valence. As such, if only higher-accuracy bias was at play then there should be no differences in the average values between groups and valences at the last generation, but the difference should manifest between their variabilities. On the other hand, ingroup-positivity bias predicts that these values should diverge, with positive traits becoming more frequent and negative traits less frequent for ingroup, as compared to the outgroup (and neutral traits staying at the same level in both cases).

Second, in our study we decided to focus primarily on psychological traits representing three types of emotional valence. It remains an open question how the biases discovered in our study would affect other types of traits or group attributes. This question is especially interesting in the context of our results showing high diversity of effects between traits, even if they were representing the same valence. It suggests that there might be other factors than valence affecting group evaluation and biasing the transmission of information about it. This is in line with the findings that ingroup bias is not a homogenous phenomenon nut rather that there are multiple types of ingroup biases^16,30^ which differently manifest themselves in different tasks and for different types of information^29,92^.

Finally, future studies could test how the transmission of information about real groups compares to the minimal group paradigm. Familiarity is a powerful factor that affects processing of information about ingroup and other self-related information^61,93–96^. This is especially true regarding group membership where the history of interaction between groups, often involving competition or cooperation, can strongly influence the behavior of involved individuals^97–99^. Moreover, minimal group paradigm allows to form an extremely minimal form of social identity, so comparing the effects using it to the effects observed with real social identities is a necessary and natural next step.

## Conclusions

We investigated whether repeated transmission of positive, neutral, and negative information about minimal groups (ingroup and outgroup) biases the content of this information. We found that arbitrary assignment of participants into minimal ingroup and outgroup can lead to systematic biases in information transmission about these groups leading to retention of more positive and neutral traits describing an ingroup. We did not find a significant difference in respect to the negative traits, although we did find such effect at the level of some individual traits (corrupt, cowardly, and a trend for dishonest). We speculate that this pattern of results comes as a consequence of two types of ingroup-biases. First, that transmission of information about one’s ingroup is characterized by higher accuracy when transmitting information about one’s ingroup and second by an ingroup-positivity bias, manifested by the tendency to retain more positive and less negative information about the ingroup. While in our study these two biases cancelled each other out in regard to negative traits, they worked in the same direction in regard to positive and neutral ones.

Our study provides the first step towards understanding how ingroup bias can affect the processes of cultural evolution. Perhaps most importantly, our results can shed light onto how group polarization emerges and develops^25,26,100^ as they show how small ingroup-biases can accumulate over time. It would be interesting to investigate in future research under what circumstances small ingroup-biases in information transmission can snowball into large and persistent differences, potentially driving radical intergroup behavior.

## Supplementary Information

Below is the link to the electronic supplementary material.

Supplementary Material 1

Supplementary Material 2

Supplementary Material 3

Supplementary Material 4

Supplementary Material 5

## Data Availability

Data, analysis scripts and materials are available under the following link: [https://osf.io/azpcy](https:/osf.io/azpcy/?view_only=fd7d378cb00e469caa285714a865e083).
