# Supplementary material for: Biases in cultural transmission of information about a minimal ingroup
Source: Sci Rep. 2026 Jan 9;16:4959. doi: 10.1038/s41598-026-35241-x (PMC12876853; doi:10.1038/s41598-026-35241-x)
Supplement: Supplementary file 1 — Supplementary Material 1 [file 41598_2026_35241_MOESM1_ESM.pdf]

SUPPLEMENTARY MATERIALS S1

Results of the Pre-study: ratings of 72 traits

In order to select the traits to be used in the transmission chains experiment we conducted a study in which we asked 26 participants from Prolific to rate 72 preselected traits on a scale from 1 (very negative) to 7 (very positive). The results are presented in table S1.1a and S1.1b, grouped by an initial classification into four categories: negative, positive, neutral, and ambivalent.

Tab. S1*. Evaluation of 72 traits on a scale from 1 (very negative) to 7 (very positive).*

**NEGATIVE**			**POSITIVE**		
**TRAIT**	**MEAN**	**SD**	**TRAIT**	**MEAN**	**SD**
Antipathic	2.32	1.03	Attractive	5.54	1.17
Antisocial	2.54	1.17	Charismatic	6	0.8
Cold	2.73	1.00	Courageous	6	0.98
Corrupt	1.39	0.64	Creative	6.15	0.73
Cowardly	2.08	0.93	Decisive	5.58	1.07
Dependent	2.92	1.32	Empathetic	6.08	0.84
Dishonest	1.46	0.58	Friendly	6.31	0.62
Hostile	2.04	1.40	Generous	5.81	0.94
Impolite	1.62	0.57	Hardworking	6.35	0.69
Lazy	2.04	0.93	Healthy	6.35	0.94
Non-charismatic	2.73	1.00	Honest	6.35	0.75
Sick	2.39	1.20	Honorable	6.27	0.72
Stingy	2.5	0.81	Independent	6.08	0.63
Stupid	1.81	0.69	Intelligent	6.42	0.58
Ugly	2.65	1.23	Polite	6.19	0.98
Unattractive	2.81	1.17	Pretty	5.15	1.38
Undecided	2.69	1.01	Prosocial	5.04	1.25
Unimaginative	2.5	0.91	Skillful	6.31	0.79
Unskilled	2.19	0.85	Sympathetic	5.85	0.88
Without empathy	1.96	0.87	Warm	5.96	0.66

Tab. S1 (continued)*. Evaluation of 72 traits on a scale from 1 (very negative) to 7 (very positive).*

NEUTRAL			AMBIVALENT		
**TRAIT**	**MEAN**	**SD**	**TRAIT**	**MEAN**	**SD**
Ambitious	5.27	1.31	Extreme	3.73	1.61
Busy	4.04	1.15	Mystical	4.19	0.98
Calm	5.96	0.82	Old	3.65	1.02
Changing	4.15	1.22	Political	3.62	1.44
Competitive	4.81	1.39	Conservative	3.62	1.60
Cooperative	6.12	0.82	Liberal	5	1.41
Dreamy	4.5	1.03	Moderate	4.81	0.75
Extraverted	4.35	1.29	Progressive	5.46	1.56
Familiar	5.27	1.15	Radical	3.15	1.41
Formal	4.54	1.03	Religious	3.54	1.70
Intense	4.46	0.90	Spiritual	4.19	1.47
Introverted	3.81	1.20	Young	4.65	0.85
Modern	5.08	1.09			
Predictable	4.23	1.34			
Private	4.96	1.11			
Sensual	4.96	1.25			
Shy	3.5	0.95			
Stable	5.92	1.02			
Traditional	4.35	1.33			
Trendy	4.35	1.26			

Demographic information about the participants

Demographics from one person were not available. Out of the remaining 25: 13 declared as females and 12 as males; mean age was 32.3 years (*SD*=13.3, min=21, max=70), nationality: United Kingdom (5), Poland (4), Hungary (3), Greece, Spain, Italy, Portugal, South Africa, Mexico (2), DATA_EXPIRED (1).
