# Supplementary material for: Biases in cultural transmission of information about a minimal ingroup
Source: Sci Rep. 2026 Jan 9;16:4959. doi: 10.1038/s41598-026-35241-x (PMC12876853; doi:10.1038/s41598-026-35241-x)
Supplement: Supplementary file 2 — Supplementary Material 2 [file 41598_2026_35241_MOESM2_ESM.pdf]

SUPPLEMENTARY MATERIALS S2

Results of control experiment 1: beliefs about the percentage of occurrence of traits in general population

Control experiment 1 investigated what are the beliefs about the percentage of occurrence of traits used in the transmission chain experiment. 51 people completed this study on Prolific. Figure S2.1 demonstrates a single trial and table S2.1 presents the results.

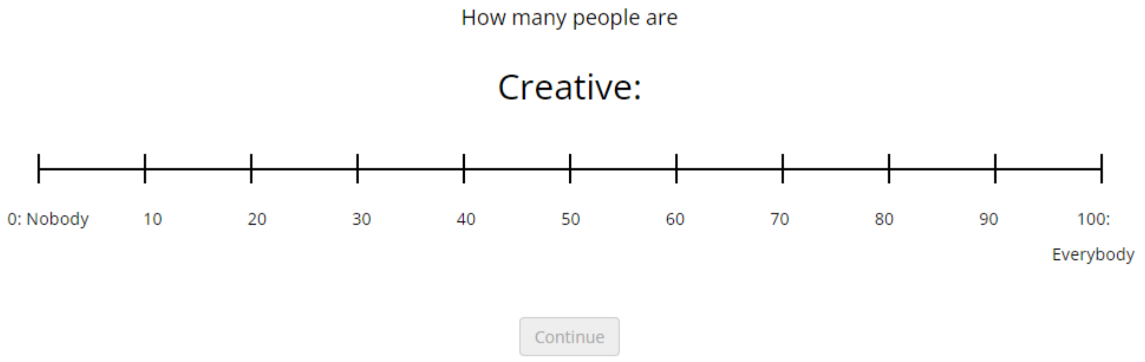

Fig. S1. *An example of a single trial.*

Tab. S2 *Beliefs about percentage of occurrence of traits in general population (mean, standard deviation, 25th percentile, median, 75th percentile).*

Trait	Mean	Sd	25%	50%	75%
**POSITIVE**					
Friendly	64.06	16.08	54	65	76
intelligent	53.41	17.79	40	50	69
honorable	48.75	16.11	38	50	60
Skilful	57.22	16.39	50	59	70
charismatic	46.45	16.4	35.5	50	55
Creative	52.75	18.73	40	55	66
**NEUTRAL**					
Trendy	48.94	16.15	37.5	46	61
Busy	67.27	15.91	55	70	78.5
traditional	53.04	18.89	40	50	70
predictable	65.04	15.53	50	65	79
introverted	49.84	12.8	42.5	50	57.5
Mystical	32.14	18	20	30	41.5
**NEGATIVE**					
corrupt	43.12	24.22	25.5	36	60
dishonest	49.1	22.2	31	45	65
lazy	52.55	17.22	39.5	50	70
without empathy	39.45	21.91	20.5	34	59.5
impolite	44.25	18.3	31.5	44	54
cowardly	51.57	16.97	40	57	61
**ADDITIONAL**					
Attractive	54.45	20.85	40	54	70
Political	52	19.92	36.5	52	68.5
Religious	50.96	19.64	36	50	64

Tab. S3. *Results of the comparison between the believed occurrence of traits in general population (Mean Occurr.) from our Control Experiment 1 and the percentage of occurrence of these traits at generation 10 in the transmission chains experiment (Mean Gen10). The "Sign." column indicates whether the comparison was statistically significant.*

Trait name	t-value	df	*p*	Sign.	Cohen's d	Mean Occurr.	Mean Gen10	Difference
**POSITIVE**								
Friendly	6.979	67	.000	Yes	-0.26	64.06	34.90	-29.16
Intelligent	3.214	67	.003	Yes	-0.11	53.41	39.52	-13.89
Honorable	3.974	67	.000	Yes	-0.16	48.75	30.13	-18.61
Skillful	5.453	67	.000	Yes	-0.18	57.22	36.06	-21.15
Charismatic	2.859	67	.008	Yes	-0.12	46.45	32.83	-13.62
Creative	3.628	67	.001	Yes	-0.12	52.75	37.11	-15.63
**NEUTRAL**								
Trendy	3.997	67	.000	Yes	-0.15	48.94	31.92	-17.02
Busy	6.023	67	.000	Yes	-0.24	67.27	40.41	-26.86
Traditional	4.866	67	.000	Yes	-0.17	53.04	30.34	-22.70
Predictable	6.109	67	.000	Yes	-0.22	65.04	41.18	-23.86
Introverted	4.447	67	.000	Yes	-0.22	49.84	29.38	-20.46
Mystical	-2.083	67	.042	Yes	0.06	32.14	39.50	7.36
**NEGATIVE**								
Corrupt	1.998	67	.051	No	-0.05	43.12	33.92	-9.19
Dishonest	3.291	67	.002	Yes	-0.11	49.10	31.88	-17.21
Lazy	3.927	67	.001	Yes	-0.16	52.55	32.77	-19.78
Without empathy	-0.600	67	.552	No	0.02	39.45	42.32	2.87
Impolite	3.592	67	.001	Yes	-0.11	44.25	30.05	-14.21
Cowardly	2.966	67	.005	Yes	-0.10	51.57	39.23	-12.33
**ADDITIONAL**								
Attractive	2.888	67	.006	Yes	-0.08	54.45	42.47	-11.98
Political	3.410	67	.002	Yes	-0.15	52.00	31.02	-20.98
Religious	3.927	67	.000	Yes	-0.09	50.96	37.81	-13.15

Demographic information about the participants

Data from one participant is not available. Among the remaining 50 participants 25 declared as females and 25 as males. Their mean age was 26.4 years (*SD*=6.6, min=19, max=51). Nationality: Portugal (11), South Africa (9), Poland (8), Mexico (5), Italy (3), Spain, Hungary (2), Chile, Switzerland, Canada, Belgium, United Kingdom, United States, Netherlands, Algeria, Zimbabwe (1), DATA_EXPIRED (1)
