# Supplementary material for: Biases in cultural transmission of information about a minimal ingroup
Source: Sci Rep. 2026 Jan 9;16:4959. doi: 10.1038/s41598-026-35241-x (PMC12876853; doi:10.1038/s41598-026-35241-x)
Supplement: Supplementary file 3 — Supplementary Material 3 [file 41598_2026_35241_MOESM3_ESM.pdf]

SUPPLEMENTARY MATERIALS S3

Results of control experiment 2: investigating response bias in a number-to-position task

Control experiment 2 investigated whether performing a simple number-to-position task leads to a similar response bias to the one that we observed in our transmission chains experiment. 30 participants completed this study on Prolific. It consisted of 99 trials in which participants were presented with all numbers between 1% and 99% and were told to indicate this number on a line. The numbers were presented in random order. Figure S3.1 demonstrates a single trial.

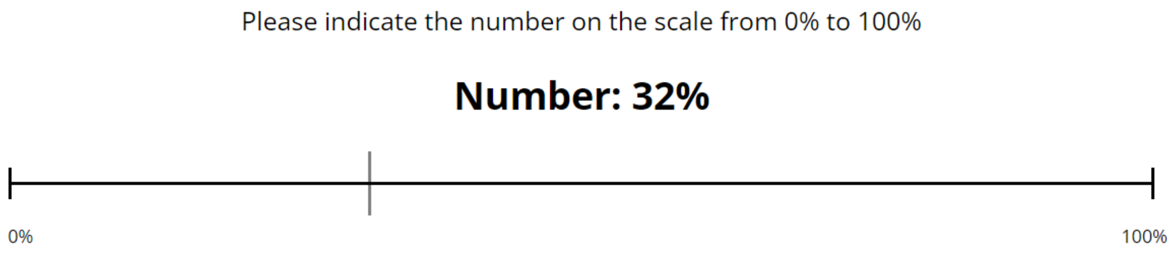

Fig. S2. *An example of a single trial.*

Tab. S4*. Descriptive statistics of the average difference between a given number and participants' responses in Control Experiment 2 (mean, standard deviation, 25th percentile, median, 75th percentile) for bins every 10 points.*

RANGE	Average difference	SD	25%	50%	75%
1-10	-1.69	2.06	-2.89	-1.44	-0.44
11-20	-3.65	3.73	-6.14	-3.22	-1.22
21-30	-4.50	4.61	-7.81	-3.67	-1.11
31-40	-6.39	5.73	-9.78	-5.33	-2.11
42-50	-2.43	5.56	-3.56	-0.78	0.56
51-60	-0.87	2.17	-2	-0.78	0.36
61-70	-0.58	4.78	-3.11	-0.78	1.67
71-80	0.89	4.58	-1.56	0.89	3.72
81-90	-0.17	4.02	-2.61	0.39	2.56
91-99	0.05	2.49	-0.78	0.33	1.33

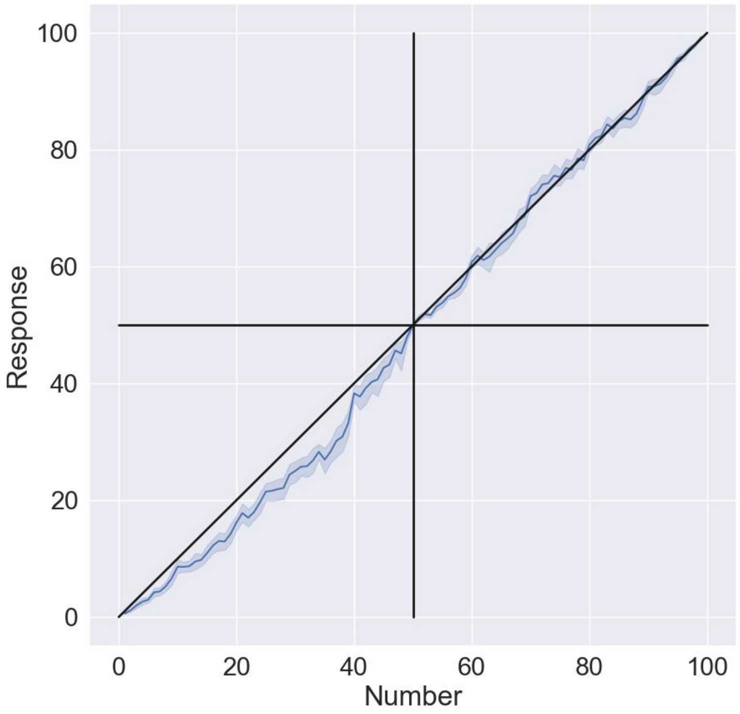

Fig. S3. *Plot of participants' responses (y-axis) on a line scale as a function of the number that they had to indicate (x-axis).*

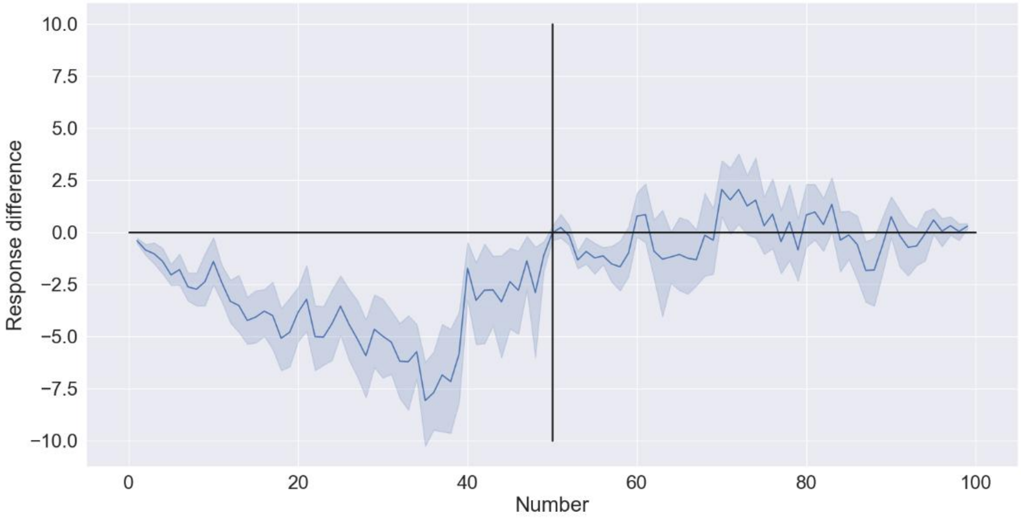

Fig. S4. *Average deviation of participants' responses from the target number. The outline indicates 95% confidence interval.*

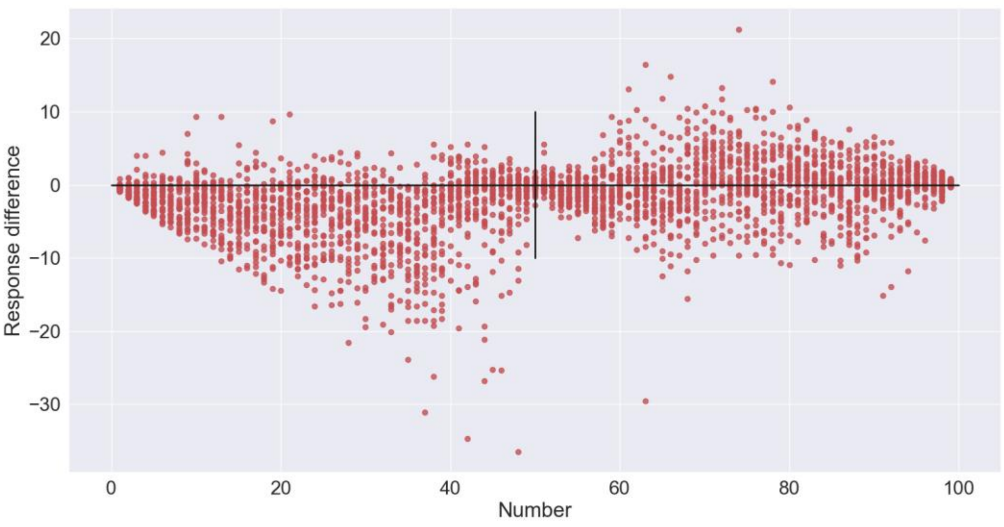

Fig. S5. *Scatterplot of deviations of participants' responses from the target number.*

Response bias in a number-to-position task in the transmission chain experiment

Tables S3.2a and S3.2b display average bias in the transmission chain experiment, separately for ingroup and outgroup data.

Tab. S5. *Descriptive statistics of the average difference between a given number and participants' responses when reproducing ingroup information in the transmission Chain Experiment (mean, standard deviation, 25th percentile, median, 75th percentile) – divided into traits describing ingroup and outgroup.*

INGROUP						
**RANGE**	**N**	**Average difference**	**SD**	**25%**	**50%**	**75%**
1-10	196	0.30	7.87	-1.92	-0.67	0.14
11-20	285	-0.60	9.21	-4.67	-2.00	0.44
21-30	350	-2.14	9.53	-6.89	-2.89	0
31-40	436	-3.30	7.07	-7.00	-3.71	-0.44
42-50	752	-0.97	4.69	-2.89	-0.67	0.89
51-60	1163	-0.86	5.23	-1.78	-0.33	0.89
61-70	449	-1.61	6.23	-3.89	-0.89	1.78
71-80	128	-0.66	7.24	-2.97	0.83	3.46
81-90	21	-0.42	3.51	-1.89	0.11	1.89
91-99	0	-	-	-	-	-

Tab. S5 (continued)*. Descriptive statistics of the average difference between a given number and participants' responses when reproducing ingroup information in the transmission Chain Experiment (mean, standard deviation, 25th percentile, median, 75*[th] *percentile) – divided into traits describing ingroup and outgroup.*

OUTGROUP						
**RANGE**	**N**	**Average difference**	**SD**	**25%**	**50%**	**75%**
1-10	206	-0.39	3.87	-1.89	-0.78	0.22
11-20	287	-1.23	8.66	-5.44	-2.22	0.33
21-30	410	-2.34	7.15	-6.00	-2.54	0.53
31-40	494	-2.76	7.08	-6.89	-3.39	0
42-50	702	-1.10	5.35	-3.08	-0.78	0.89
51-60	1124	-1.15	5.83	-2.00	-0.44	1
61-70	448	-2.57	8.76	-4.22	-1.26	1.89
71-80	96	-2.28	10.79	-3.42	-0.28	2.69
81-90	13	-0.40	3.23	-2.11	0	1.67
91-99	0	-	-	-	-	-

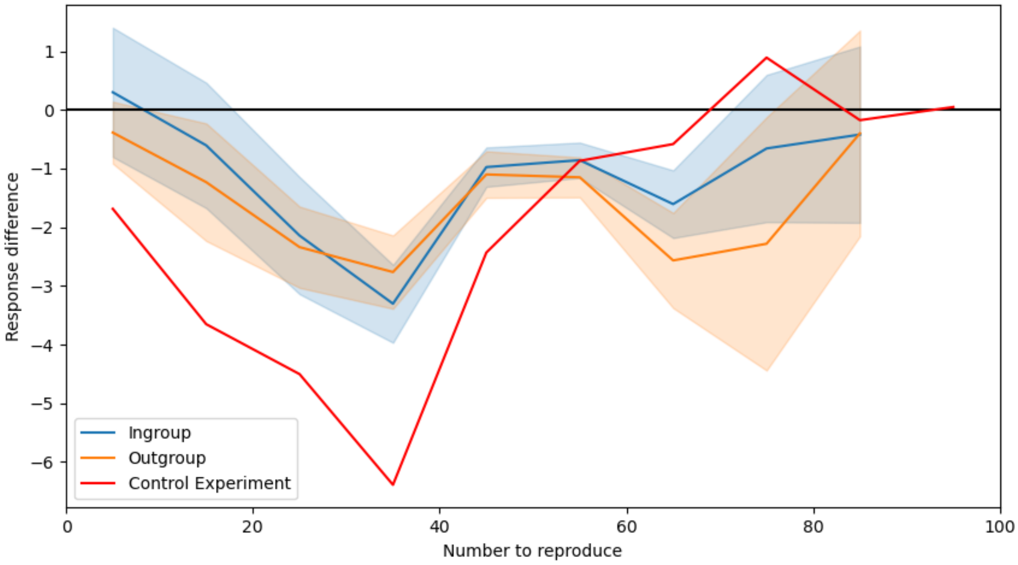

Fig. S6. *Average deviation of participants' responses from the target number from the transmission chains study, separately for ingroup and outgroup data. The averages were calculated for blocks of 10 points (from 0% to 10%, from 11% to 20% etc.). The outline indicates 95% confidence interval. The black line indicates a situation in which there would be no bias. The red line illustrates the results of Control Experiment 2 showing that in the 0%-50% range the response bias was larger in the control experiment.*

Demographic information about the participants

Data from one participant is not available. Among the remaining 30 participants 15 declared as females and 15 as males. Their mean age was 27.9 years (*SD*=10.6, min=19, max=77). Nationality: South Africa (8), Portugal (5), UK, Italy, Mexico (3), Poland, Hungary (2), Vietnam, Costa Rica, Spain (1), DATA_EXPIRED (1)
