# Supplementary material for: Biases in cultural transmission of information about a minimal ingroup
Source: Sci Rep. 2026 Jan 9;16:4959. doi: 10.1038/s41598-026-35241-x (PMC12876853; doi:10.1038/s41598-026-35241-x)
Supplement: Supplementary file 4 — Supplementary Material 4 [file 41598_2026_35241_MOESM4_ESM.pdf]

SUPPLEMENTARY MATERIALS S4

Results of the preregistered analyses of transmission chains

This supplementary material reports the results of all preregistered analyses. We only report the results of preregistered analyses 1 and 2 here, because the results of the full preregistered analysis 3 are reported in the manuscript.

Design

The experiment involved three preregistered analyses:

Analysis 1: RQ1 and RQ2 will be answered through a 2-way repeated-measures ANOVA with factors: group (Ingroup vs. Outgroup) and valence (negative vs. neutral vs. positive) comparing:

a) Transmitted frequencies of occurrences for the last elements of the transmission chain.
b) Slopes of functions fitted to transmission chains across generations

Analysis 2: A 3-way mixed effects ANOVA with factors: group (within-subject: Ingroup vs. Outgroup), valence (within-subject: negative vs. neutral vs. positive), and generation (1 to 10) on transmitted frequencies of occurrences

The exact two analyses will be also conducted using a mixed models approach, with transmission chains added as random effects

Analysis 3: A one-sample t-test comparison between frequencies of occurrences at generation 10 for each trait and average believed frequencies of occurrence of these traits in general population (estimated in a control study)

Results

Preregistered analysis 1a. A preregistered 2-way ANOVA with factors Valence (positive vs. neutral, vs. negative) and Group (ingroup vs. outgroup) in PO (percentage of trait occurrence) values at generation 10 revealed a marginally significant main effect of group ($F(1, 17)=4.2$, $p=.057$, partial $\eta^2=.20$) reflecting that average POs for ingroup ($M=36.7\%$) were higher than for outgroup ($M=33.7\%$). The main effect of valence ($F(2, 34)=.03$, $p=.97$, partial $\eta^2<.01$) and the interaction effect ($F(2, 34)=1.23$, $p=.31$, partial $\eta^2=.07$) were not significant. Figure S4.1 illustrates the main effect of group.

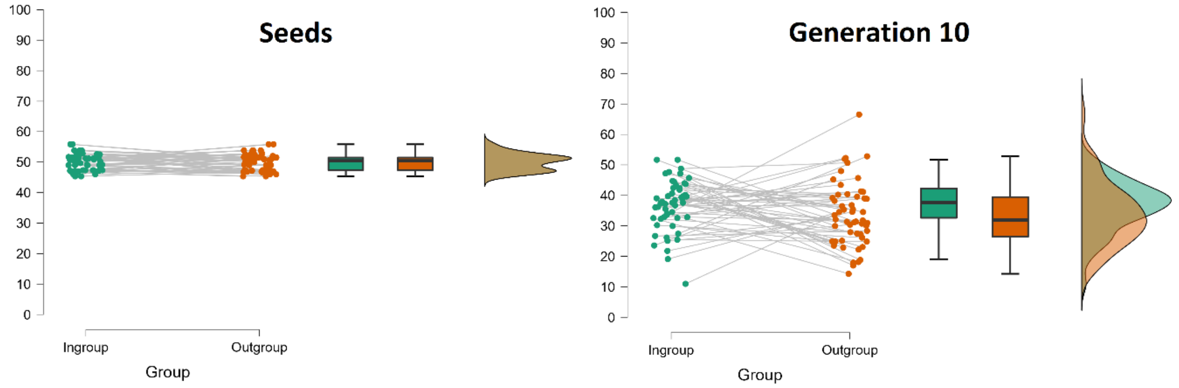

Figure S7. *Percentage of occurrence of traits (PO) at seed values (left) and at the last generation (right) for traits of all valences (positive, neutral and negative). Information about both groups showed a strong decrease in POs, but this decrease was stronger for outgroup than ingroup traits.*

Preregistered analysis 1b. This has been investigated in the preregistered analysis 2 with Linear Mixed Models approach.

Preregistered analysis 2. A 3-way ANOVA treating Generation as a factor and not as a covariate. It means that this analysis does not take into account the fact that Generation was a continuous variable, but rather treats each generation as qualitatively different from each other. Such treatment can be justified when the time course of effects across generations is highly nonlinear.

A preregistered 3-way ANOVA with factors Valence (positive vs. neutral, vs. negative), Group (ingroup vs. outgroup), and Generation (0 to 10) for the PO values revealed a significant main effect of generation ($F(10, 187)=39.8$, $p<.001$, partial $\eta^2=.68$), a significant main effect of group ($F(1, 187)=9.23$, $p=.003$, partial $\eta^2=.05$), a significant main effect of

valence (F(1.72, 321.8)=5.28, p=0.008 Greenhouse-Geisser corrected, partial η^2=.03), and a significant interaction between group and valence (F(1.78, 332.5)=9.2, p<.001 Greenhouse-Geisser corrected, partial η^2=.05). The remaining effects were not significant: the interaction between group and generation (F(10,187)=1.19, p=.30, partial η^2=.06), the interaction of valence and generation (F(17.2, 321.8)=.2, p=1 Greenhouse-Geisser corrected, partial η^2=.01), and the 3-way interaction (F(17.8, 332.5)=.4, p=0.99, partial η^2=.02). The main effect of generation reflected a generalized reduction in PO values across generations, and the main effect of groups showed overall significantly lower PO values for outgroup than ingroup.

In order to further investigate the interaction between group and valence we conducted post hoc tests comparing POs for ingroup vs. ingroup for each of the three trait valences. The results revealed that the difference between groups was significant for positive (t=3.29, p<.001, all Bonferroni corrected, Cohen's d=.32), and neutral traits (t=3.21, p=.006, Cohen's d=.32) indicating higher PO values for ingroup. For the negative traits POs were lower for ingroup than outgroup, but the difference was only marginally significant (t=2.55, p=.054, Cohen's d=.25). Figure 3 illustrates the results of the analysis.

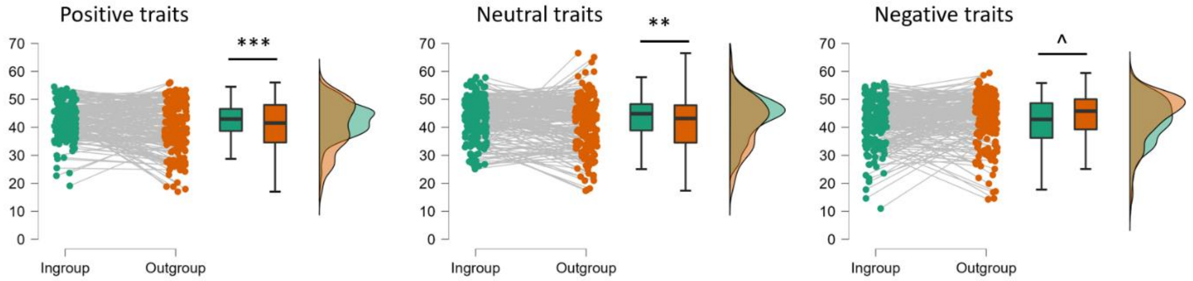

Figure S8. *Percentage of occurrence of traits (PO) across all generations in ingroup and outgroup, separately for positive, neutral, and negative traits. *** p<.001, ** p<.01, ^ p<.1*

Preregistered analysis 2 with Linear Mixed Models (extended report of the analysis described in the manuscript): To answer research questions 1 and 2 we conducted a linear mixed effects model analysis with factors Group (Ingroup vs. Outgroup) and Valence (Positive vs. Neutral vs. Negative), Generation treated as a covariate and participants and chains treated as random factors. Because the full model did not converge we report results of the model with only chains as random factors. We allowed the chains to have both random intercepts and random slopes (as a function of generation). The analyses were run in R (v4.3.0) using the lme4 package (v.1.1-33).

The results of the model (AIC = 7726.83, BIC = 7808.11, Pseudo-R^2 (fixed effects) = .35, Table 1) revealed a significant main effect of Generation reflecting a systematic decline of values across generations. An interaction between Group and Valence was significant when contrasted against the negative valence, and was marginally significant when contrasted against the positive valence. However, these two-way interactions were qualified by a three-way interaction between Group, Valence and Generation, which was significant for negative valence as a reference category, and marginally significant for positive valence as a reference category. The remaining effects were not significant.

Table S6*. The results of the full linear mixed model. All p-values calculated with Satterthwaite method.*

Three-way linear mixed model

MODEL FIT:
AIC = 7726.83, *BIC* = 7808.11
Pseudo-R^2 (fixed effects) = 0.35
Pseudo-R^2 (total) = 0.47

FIXED EFFECTS:

	Est.	S.E.	t val.	d.f.	p
(Intercept)	50.03	0.83	60.04	282.10	.000
GroupOu	1.05	1.14	0.93	1142.00	.353
Generation	-1.60	0.17	-9.54	80.74	.000
ValenceNeg	-0.48	1.14	-0.43	1142.00	.671
ValencePos	-1.11	1.14	-0.98	1142.00	.329
GroupOu:Generation	0.13	0.19	0.66	1142.00	.508
GroupOu:ValenceNeg	-0.17	1.61	-0.11	1142.00	.914
GroupOu:ValencePos	-1.41	1.61	-0.88	1142.00	.379
Generation:ValenceNeg	0.40	0.19	2.07	1142.00	.039
Generation:ValencePos	0.33	0.19	1.72	1142.00	.085
GroupOu:Generation:ValenceNeg	-0.73	0.27	-2.69	1142.00	.007
GroupOu:Generation:ValencePos	-0.49	0.27	-1.81	1142.00	.070

RANDOM EFFECTS:

Group	Parameter	Std. Dev.
Chain_id	(Intercept)	0.95
Chain_id	Generation	0.42
Residual		6.04

Grouping variables:

Group	# groups	ICC
Chain_id	18	0.02

In order to further explain the three-way interaction we conducted additional analyses separately for data of each valence (see Supplementary Materials S4 for full results and Figure 2 for illustration of the results). In each case it was a linear mixed model with Group as a factor, Generation as a covariate and chains as a random effect with random slope and

random intercept (the inclusion of participants as a random effect was not justified as indicated by a Chi2 test, p=1). For positive traits the model (AIC = 2413.43, BIC = 2445.28, Pseudo-R^2 (fixed effects) = .39) revealed a significant main effect of generation ($t(32.4)=-8.66$, $p<.001$) and a significant interaction between group and generation ($t(360)=-2.46$, $p=.014$). The main effect of group was not significant ($t(360)=.41$, $p=.684$). The significant interaction reflected stronger negative slope for outgroup than for ingroup.

The same pattern of results was present for neutral traits (AIC = 2496.65, BIC = 2528.50, Pseudo-R^2 (fixed effects) = .35): a significant main effect of generation ($t(32.33)=-7.41$, $p<.001$) and a significant interaction between group and generation ($t(360)=-3.67$, $p<.001$), with non-significant main effect of group ($t(360)=.91$, $p=.365$).

For negative traits (AIC = 2537.63, BIC = 2569.48, Pseudo-R^2 (fixed effects) = .33) the main effect of generation was significant ($t(29.22)=-8.59$, $p<.001$), while the main effect of group ($t(360)=1.03$, $p=.305$) and the interaction ($t(360)=.73$, $p=.464$) were not.

Table S7*. The results of the linear mixed model for positive valence. All p-values calculated with Satterthwaite method.*

POSITIVE

MODEL FIT:
AIC = 2416.48, *BIC* = 2448.33
Pseudo-R^2 (fixed effects) = 0.38
Pseudo-R^2 (total) = 0.63

FIXED EFFECTS:

	Est.	S.E.	t val.	d.f.	p
(Intercept)	48.92	0.77	63.92	37.50	.000
GroupOu	-0.36	0.88	-0.41	358.00	.684
Generation	-1.27	0.15	-8.47	29.80	.000
GroupOu:Generation	-0.36	0.15	-2.45	358.00	.014

RANDOM EFFECTS:

Group	Parameter	Std. Dev.
Chain_id	(Intercept)	1.89
Chain_id	Generation	0.45
Residual		4.68

Grouping variables:

Group	# groups	ICC
Chain_id	18	0.14

Table S8. *The results of the linear mixed model for neutral valence. All p-values calculated with Satterthwaite method.*

NEUTRAL

MODEL FIT:
AIC = 2498.71, *BIC* = 2530.56
Pseudo-R² *(fixed effects)* = 0.34
Pseudo-R² *(total)* = 0.63

FIXED EFFECTS:

	Est.	S.E.	t val.	d.f.	p
(Intercept)	49.54	0.90	55.03	33.67	.000
GroupOu	0.88	0.97	0.91	358.00	.365
Generation	-1.20	0.17	-7.25	29.78	.000
GroupOu:Generation	-0.60	0.16	-3.66	358.00	.000

RANDOM EFFECTS:

Group	Parameter	Std. Dev.
Chain_id	(Intercept)	2.46
Chain_id	Generation	0.50
Residual		5.18

Grouping variables:

Group	# groups	ICC
Chain_id	18	0.18

Table S9. *The results of the linear mixed model for negative valence. All p-values calculated with Satterthwaite method.*

NEGATIVE

MODEL FIT:
AIC = 2539.40, *BIC* = 2571.25
Pseudo-R² *(fixed effects)* = 0.32
Pseudo-R² *(total)* = 0.60

FIXED EFFECTS:

	Est.	S.E.	t val.	d.f.	p
(Intercept)	50.03	0.85	59.02	41.91	.000
GroupOu	1.05	1.03	1.03	358.01	.305
Generation	-1.60	0.19	-8.39	27.01	.000
GroupOu:Generation	0.13	0.17	0.73	358.01	.464

RANDOM EFFECTS:

Group	Parameter	Std. Dev.
Chain_id	(Intercept)	1.85
Chain_id	Generation	0.62
Residual		5.47

Grouping variables:

Group	# groups	ICC
Chain_id	18	0.10

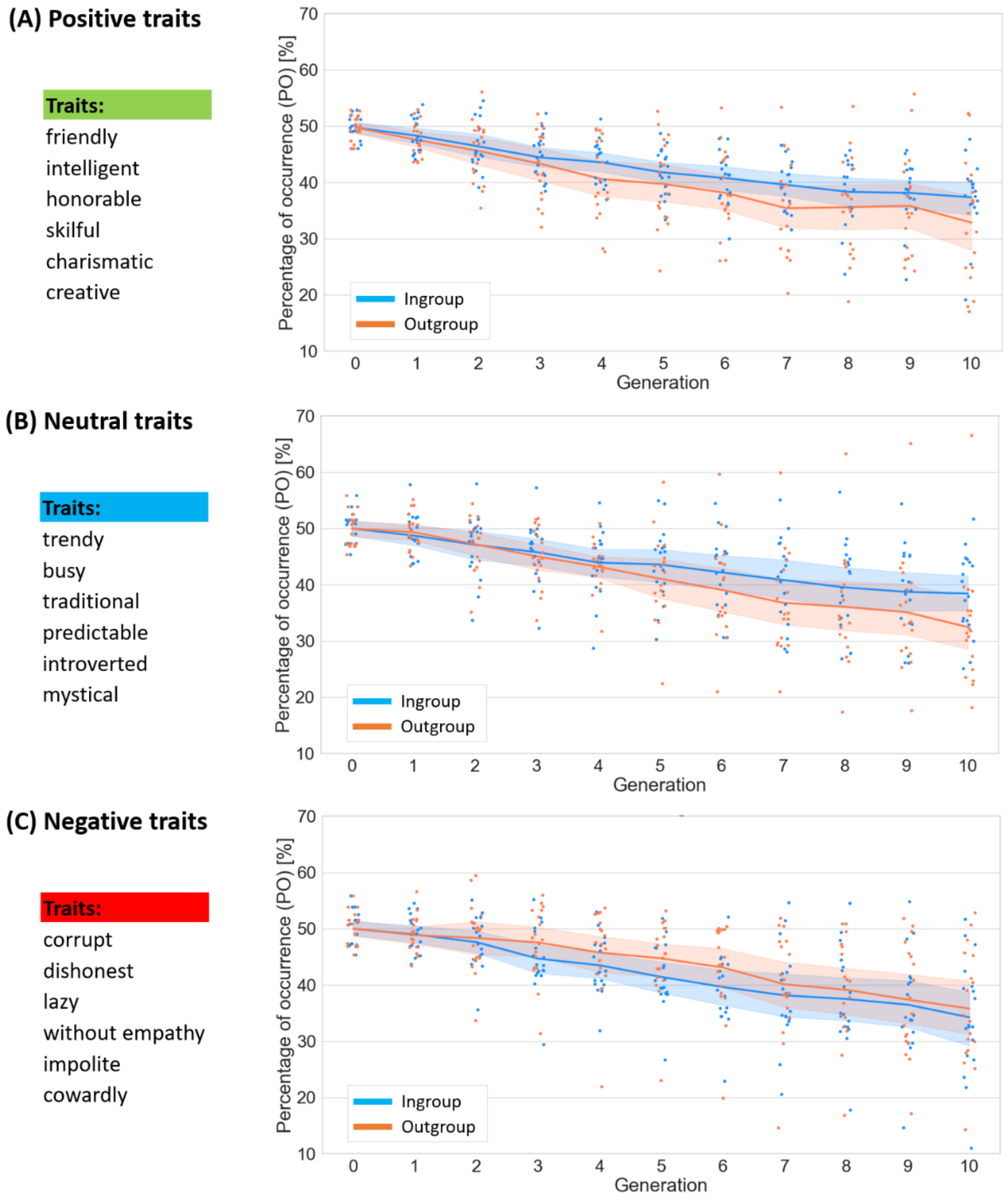

Figure S9. *Percentage of trait occurrence (POT) averaged for each valence as a function of generation.*

Additional plots comparing the results between valences for each group. For illustrative purposes we also present plots of results comparing traits of different valences within each group.

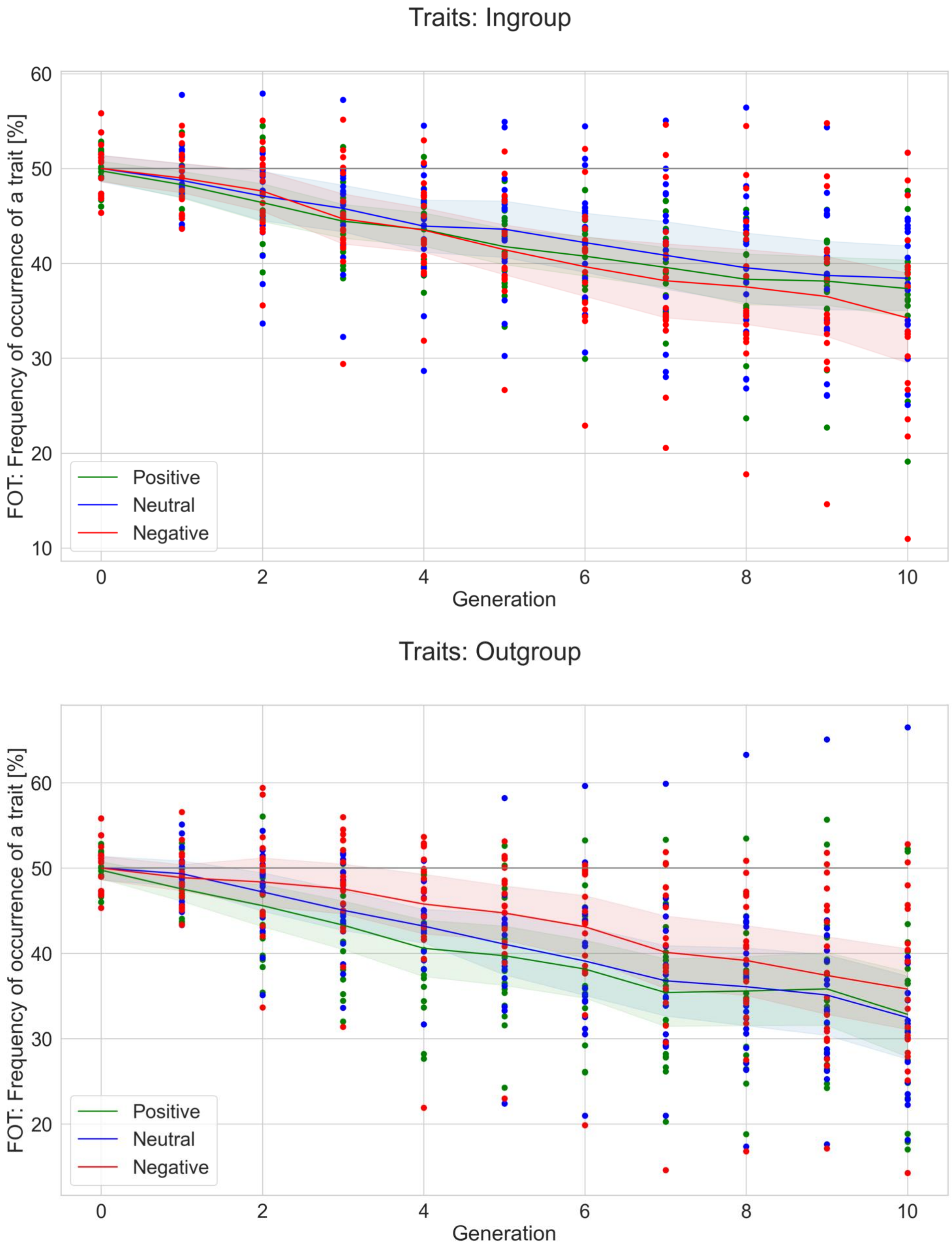

Figure S10. *Percentage of trait occurrence (POT) for each group as a function of generation and valence.*
