# Supplementary material for: Biases in cultural transmission of information about a minimal ingroup
Source: Sci Rep. 2026 Jan 9;16:4959. doi: 10.1038/s41598-026-35241-x (PMC12876853; doi:10.1038/s41598-026-35241-x)
Supplement: Supplementary file 5 — Supplementary Material 5 [file 41598_2026_35241_MOESM5_ESM.pdf]

SUPPLEMENTARY MATERIALS S5

Results and discussion of the results of transmission chains for individual traits

When inspecting individual traits, our results generally confirm the finding that valence affected how group membership influenced the trajectories of cultural evolution, but also showed that this effect was highly variable across different traits. Interested readers can explore these results in an interactive dashboard available at the following address: https://mmwozniak-cultevoself.streamlit.app/

For positive traits, a significant interaction between groups and generation in the direction predicted by the ingroup-positivity bias (slower decrease for ingroup than outgroup) was present for traits “friendly”, and “intelligent”. This effect was marginally significant for the trait “creative” and not significant for “charismatic” and “honorable”. The results for the trait “skillful” come as a surprise, as they demonstrated significantly faster decrease in their POs for ingroup than outgroup – an effect expected for a negative trait but not for a positive one. This result is especially surprising given that even neutral traits showed a strong tendency for slower decrease in ingroup than outgroup. Future research should determine whether it was just a spurious result or whether skillfulness is indeed treated in a special manner in the context of group-description.

For negative traits, the interaction effect between group and generation was significant for the traits “corrupt” and “cowardly”, and marginally significant for “dishonest” reflecting faster decrease of POs for ingroup than outgroup. For the traits “lazy”, “impolite” and "without empathy” the interaction was not significant. These results show that while on average negative traits did not show evidence of higher ingroup-positivity, such bias was present for some individual traits. This finding provides additional support for our interpretation that people display favoritism towards their minimal ingroup by transmitting less negative information. However, this effect is either more limited (only to specific traits) or overall weaker in regard to negative traits than in regard to positive traits. As we proposed earlier this might be due to the presence of a second type of bias: the general motivation to be more accurate when transmitting information about one’s ingroup that partially (but not fully) cancels out the ingroup-positivity bias in negative traits.

Finally, for neutral traits, the difference between groups was primarily driven by three traits: busy, predictable, and mystical, while for trendy, traditional, and introverted the

difference was not significant, also showing high variability of the magnitude of effect across different traits.

Overall, our results show that while valence interacted with group membership, reflecting the ingroup-positivity bias, there was a strong variability across traits and this effect was mostly driven by specific traits showing strong susceptibility to group membership (especially traits such as: friendly, intelligent, attractive, corrupt, cowardly), and not by a small effect evenly spread out across all traits of a given valance. This high variability across traits was also present in neutral traits, where three traits (busy, predictable and mystical) strongly drove the effect for the whole category.

Results of Linear Mixed Models for individual traits

The LMM analyses for individual traits were performed in Python (and not in R), so they could be displayed in an interactive form using a dashboard. The results of these analyses are also reported below.

POSITIVE 1: FRIENDLY

```
Mixed Linear Model Regression Results
========================================================================
Model:                  MixedLM      Dependent Variable:       Average
No. Observations:       396          Method:                   REML
No. Groups:             18           Scale:                    124.4279
Min. group size:        22           Log-Likelihood:           -1542.9816
Max. group size:        22           Converged:                Yes
Mean group size:        22.0
------------------------------------------------------------------------
                              Coef.  Std.Err.   z    P>|z| [0.025 0.975]
------------------------------------------------------------------------
Intercept                     45.479    3.285 13.844 0.000 39.040 51.918
Group[T.Outgroup]              3.079    2.097  1.468 0.142 -1.032  7.190
Generation                    -0.483    0.251 -1.925 0.054 -0.974  0.009
Group[T.Outgroup]:Generation  -1.611    0.355 -4.543 0.000 -2.306 -0.916
Group Var                    154.664    5.040
========================================================================
```

POSITIVE 2: INTELLIGENT

```
Mixed Linear Model Regression Results
========================================================================
Model:                  MixedLM      Dependent Variable:       Average
No. Observations:       396          Method:                   REML
No. Groups:             18           Scale:                    163.5162
Min. group size:        22           Log-Likelihood:           -1590.9918
Max. group size:        22           Converged:                Yes
Mean group size:        22.0
------------------------------------------------------------------------
```

```
                              Coef.  Std.Err.   z    P>|z| [0.025 0.975]
------------------------------------------------------------------------
Intercept                     50.951    2.929 17.393 0.000 45.209 56.692
Group[T.Outgroup]             -0.424    2.404 -0.176 0.860 -5.136  4.288
Generation                    -0.522    0.287 -1.816 0.069 -1.085  0.041
Group[T.Outgroup]:Generation  -1.089    0.406 -2.679 0.007 -1.885 -0.292
Group Var                    102.437    3.013
========================================================================
```

POSITIVE 3: HONORABLE

```
Mixed Linear Model Regression Results
========================================================================
Model:                  MixedLM      Dependent Variable:      Average
No. Observations:       396          Method:                  REML
No. Groups:             18           Scale:                   219.9748
Min. group size:        22           Log-Likelihood:          -1649.1128
Max. group size:        22           Converged:               Yes
Mean group size:        22.0
------------------------------------------------------------------------
                              Coef.  Std.Err.   z    P>|z| [0.025 0.975]
------------------------------------------------------------------------
Intercept                     47.518    3.396 13.993 0.000 40.862 54.174
Group[T.Outgroup]             -0.784    2.789 -0.281 0.779 -6.249  4.682
Generation                    -2.057    0.333 -6.171 0.000 -2.710 -1.404
Group[T.Outgroup]:Generation   0.520    0.471  1.102 0.270 -0.404  1.443
Group Var                    137.583    3.489
========================================================================
```

POSITIVE 4: SKILLFUL

```
Mixed Linear Model Regression Results
=======================================================================
Model:                 MixedLM      Dependent Variable:      Average
No. Observations:      396          Method:                  REML
No. Groups:            18           Scale:                   160.2038
Min. group size:       22           Log-Likelihood:          -1581.1182
Max. group size:       22           Converged:               Yes
Mean group size:       22.0
-----------------------------------------------------------------------
                             Coef.  Std.Err.   z    P>|z| [0.025 0.975]
-----------------------------------------------------------------------
Intercept                    49.740    2.330 21.350 0.000 45.174 54.306
Group[T.Outgroup]             0.585    2.380  0.246 0.806 -4.080  5.249
Generation                   -2.156    0.284 -7.581 0.000 -2.714 -1.599
Group[T.Outgroup]:Generation  1.303    0.402  3.240 0.001  0.515  2.092
Group Var                    46.725    1.496
=======================================================================
```

POSITIVE 5: CHARISMATIC

```
Mixed Linear Model Regression Results
========================================================================
Model:                  MixedLM      Dependent Variable:      Average
No. Observations:       396          Method:                  REML
No. Groups:             18           Scale:                   158.8583
Min. group size:        22           Log-Likelihood:          -1587.9525
Max. group size:        22           Converged:               Yes
```

```
Mean group size:        22.0
------------------------------------------------------------------------
                              Coef.  Std.Err.   z    P>|z| [0.025 0.975]
------------------------------------------------------------------------
Intercept                     49.244    3.238 15.210 0.000 42.899 55.590
Group[T.Outgroup]             -0.938    2.370 -0.396 0.692 -5.582  3.707
Generation                    -1.478    0.283 -5.219 0.000 -2.033 -0.923
Group[T.Outgroup]:Generation  -0.510    0.401 -1.274 0.203 -1.295  0.275
Group Var                    138.138    4.045
========================================================================
```

POSITIVE 6: CREATIVE

```
Mixed Linear Model Regression Results
=======================================================================
Model:                 MixedLM      Dependent Variable:      Average
No. Observations:      396          Method:                  REML
No. Groups:            18           Scale:                   171.5942
Min. group size:       22           Log-Likelihood:          -1599.1125
Max. group size:       22           Converged:               Yes
Mean group size:       22.0
-----------------------------------------------------------------------
                             Coef.  Std.Err.   z    P>|z| [0.025 0.975]
-----------------------------------------------------------------------
Intercept                    50.573    2.842 17.795 0.000 45.003 56.143
Group[T.Outgroup]            -3.664    2.463 -1.488 0.137 -8.492  1.163
Generation                   -0.914    0.294 -3.106 0.002 -1.491 -0.337
Group[T.Outgroup]:Generation -0.801    0.416 -1.925 0.054 -1.617  0.015
Group Var                    90.791    2.639
=======================================================================
```

NEGATIVE 1: CORRUPT

```
Mixed Linear Model Regression Results
=======================================================================
Model:                 MixedLM      Dependent Variable:      Average
No. Observations:      396          Method:                  REML
No. Groups:            18           Scale:                   139.6627
Min. group size:       22           Log-Likelihood:          -1558.5184
Max. group size:       22           Converged:               Yes
Mean group size:       22.0
-----------------------------------------------------------------------
                             Coef.  Std.Err.   z    P>|z| [0.025 0.975]
-----------------------------------------------------------------------
Intercept                    49.683    2.540 19.561 0.000 44.705 54.661
Group[T.Outgroup]            -1.646    2.222 -0.741 0.459 -6.001  2.709
Generation                   -1.023    0.266 -3.851 0.000 -1.543 -0.502
Group[T.Outgroup]:Generation -0.768    0.376 -2.044 0.041 -1.504 -0.032
Group Var                    71.688    2.316
=======================================================================
```

NEGATIVE 2: DISHONEST

```
Mixed Linear Model Regression Results
========================================================================
Model:                  MixedLM      Dependent Variable:      Average
No. Observations:       396          Method:                  REML
No. Groups:             18           Scale:                   222.8907
```

```
Min. group size:        22          Log-Likelihood:         -1650.9470
Max. group size:        22          Converged:              Yes
Mean group size:        22.0
----------------------------------------------------------------------
                             Coef.  Std.Err.   z    P>|z| [0.025 0.975]
----------------------------------------------------------------------
Intercept                    51.077    3.315 15.410 0.000 44.581 57.573
Group[T.Outgroup]             2.186    2.807  0.779 0.436 -3.316  7.688
Generation                   -2.432    0.336 -7.247 0.000 -3.089 -1.774
Group[T.Outgroup]:Generation  0.832    0.474  1.754 0.079 -0.098  1.762
Group Var                   126.828    3.217
======================================================================
```

NEGATIVE 3: LAZY

```
Mixed Linear Model Regression Results
======================================================================
Model:                  MixedLM     Dependent Variable:     Average
No. Observations:       396         Method:                 REML
No. Groups:             18          Scale:                  260.6592
Min. group size:        22          Log-Likelihood:         -1682.5716
Max. group size:        22          Converged:              Yes
Mean group size:        22.0
----------------------------------------------------------------------
                             Coef.  Std.Err.   z    P>|z| [0.025 0.975]
----------------------------------------------------------------------
Intercept                    48.551    3.727 13.026 0.000 41.245 55.856
Group[T.Outgroup]             2.277    3.036  0.750 0.453 -3.673  8.227
Generation                   -1.756    0.363 -4.841 0.000 -2.467 -1.045
Group[T.Outgroup]:Generation -0.025    0.513 -0.050 0.960 -1.031  0.980
Group Var                   167.137    3.888
======================================================================
```

NEGATIVE 4: WITHOUT EMPATHY

```
Mixed Linear Model Regression Results
=====================================================================
Model:                 MixedLM     Dependent Variable:     Average
No. Observations:      396         Method:                 REML
No. Groups:            18          Scale:                  306.4383
Min. group size:       22          Log-Likelihood:         -1706.5170
Max. group size:       22          Converged:              Yes
Mean group size:       22.0
---------------------------------------------------------------------
                            Coef.  Std.Err.   z    P>|z| [0.025 0.975]
---------------------------------------------------------------------
Intercept                    52.201    3.055 17.090 0.000 46.215 58.188
Group[T.Outgroup]            -0.296    3.291 -0.090 0.928 -6.747  6.155
Generation                   -0.942    0.393 -2.395 0.017 -1.713 -0.171
Group[T.Outgroup]:Generation -0.008    0.556 -0.014 0.988 -1.098  1.082
Group Var                    70.444    1.690
=====================================================================
```

NEGATIVE 5: IMPOLITE

```
Mixed Linear Model Regression Results
=====================================================================
Model:                 MixedLM     Dependent Variable:     Average
```

```
No. Observations:       396         Method:                  REML
No. Groups:             18          Scale:                   237.4523
Min. group size:        22          Log-Likelihood:          -1656.5180
Max. group size:        22          Converged:               Yes
Mean group size:        22.0
-----------------------------------------------------------------------
                             Coef.  Std.Err.   z    P>|z| [0.025 0.975]
-----------------------------------------------------------------------
Intercept                    46.423    2.688 17.270 0.000 41.154 51.691
Group[T.Outgroup]             2.414    2.897  0.833 0.405 -3.265  8.093
Generation                   -1.581    0.346 -4.565 0.000 -2.260 -0.902
Group[T.Outgroup]:Generation -0.361    0.490 -0.737 0.461 -1.321  0.599
Group Var                    54.513    1.486
=======================================================================
```

NEGATIVE 6: COWARDLY

```
Mixed Linear Model Regression Results
=======================================================================
Model:                  MixedLM     Dependent Variable:      Average
No. Observations:       396         Method:                  REML
No. Groups:             18          Scale:                   170.0702
Min. group size:        22          Log-Likelihood:          -1593.3811
Max. group size:        22          Converged:               Yes
Mean group size:        22.0
-----------------------------------------------------------------------
                             Coef.  Std.Err.   z    P>|z| [0.025 0.975]
-----------------------------------------------------------------------
Intercept                    52.222    2.444 21.365 0.000 47.432 57.013
Group[T.Outgroup]             1.393    2.452  0.568 0.570 -3.413  6.199
Generation                   -1.862    0.293 -6.352 0.000 -2.436 -1.287
Group[T.Outgroup]:Generation  1.092    0.414  2.636 0.008  0.280  1.905
Group Var                    53.429    1.645
=======================================================================
```

NEUTRAL 1: TRENDY

```
Mixed Linear Model Regression Results
========================================================================
Model:                   MixedLM     Dependent Variable:      Average
No. Observations:        396         Method:                  REML
No. Groups:              18          Scale:                   213.4728
Min. group size:         22          Log-Likelihood:          -1642.7393
Max. group size:         22          Converged:               Yes
Mean group size:         22.0
------------------------------------------------------------------------
                              Coef.  Std.Err.   z    P>|z| [0.025 0.975]
------------------------------------------------------------------------
Intercept                     47.630    3.278 14.532 0.000 41.206 54.054
Group[T.Outgroup]              1.002    2.747  0.365 0.715 -4.383  6.386
Generation                    -1.865    0.328 -5.681 0.000 -2.509 -1.222
Group[T.Outgroup]:Generation   0.284    0.464  0.612 0.541 -0.626  1.194
Group Var                    125.448    3.244
========================================================================
```

NEUTRAL 2: BUSY

```
Mixed Linear Model Regression Results
```

```
==============================================================================
Model:                    MixedLM       Dependent Variable:       Average
No. Observations:         396           Method:                   REML
No. Groups:               18            Scale:                    174.1498
Min. group size:          22            Log-Likelihood:           -1603.9563
Max. group size:          22            Converged:                Yes
Mean group size:          22.0
------------------------------------------------------------------------------
                               Coef.  Std.Err.   z    P>|z| [0.025 0.975]
------------------------------------------------------------------------------
Intercept                      50.737    3.103 16.352 0.000 44.655 56.818
Group[T.Outgroup]               1.172    2.481  0.473 0.637 -3.691  6.036
Generation                     -0.375    0.297 -1.263 0.207 -0.956  0.207
Group[T.Outgroup]:Generation   -1.626    0.419 -3.876 0.000 -2.448 -0.804
Group Var                     117.887    3.343
==============================================================================
```

NEUTRAL 3: TRADITIONAL

```
Mixed Linear Model Regression Results
==============================================================================
Model:                    MixedLM       Dependent Variable:       Average
No. Observations:         396           Method:                   REML
No. Groups:               18            Scale:                    182.6513
Min. group size:          22            Log-Likelihood:           -1614.1539
Max. group size:          22            Converged:                Yes
Mean group size:          22.0
------------------------------------------------------------------------------
                               Coef.  Std.Err.   z    P>|z| [0.025 0.975]
------------------------------------------------------------------------------
Intercept                      47.640    3.298 14.447 0.000 41.177 54.103
Group[T.Outgroup]               0.909    2.541  0.358 0.721 -4.072  5.889
Generation                     -2.039    0.304 -6.712 0.000 -2.634 -1.443
Group[T.Outgroup]:Generation    0.253    0.430  0.589 0.556 -0.589  1.095
Group Var                     137.618    3.786
==============================================================================
```

NEUTRAL 4: PREDICTABLE

```
Mixed Linear Model Regression Results
=============================================================================
Model:                   MixedLM       Dependent Variable:       Average
No. Observations:        396           Method:                   REML
No. Groups:              18            Scale:                    116.8483
Min. group size:         22            Log-Likelihood:           -1521.4336
Max. group size:         22            Converged:                Yes
Mean group size:         22.0
-----------------------------------------------------------------------------
                              Coef.  Std.Err.   z    P>|z| [0.025 0.975]
-----------------------------------------------------------------------------
Intercept                     51.832    2.143 24.181 0.000 47.631 56.033
Group[T.Outgroup]              1.455    2.032  0.716 0.474 -2.529  5.439
Generation                    -0.325    0.243 -1.337 0.181 -0.801  0.151
Group[T.Outgroup]:Generation  -1.421    0.344 -4.137 0.000 -2.095 -0.748
Group Var                     45.520    1.649
=============================================================================
```

NEUTRAL 5: INTROVERTED

```
Mixed Linear Model Regression Results
========================================================================
Model:                   MixedLM      Dependent Variable:      Average
No. Observations:        396          Method:                  REML
No. Groups:              18           Scale:                   215.9044
Min. group size:         22           Log-Likelihood:          -1647.5573
Max. group size:         22           Converged:               Yes
Mean group size:         22.0
------------------------------------------------------------------------
                              Coef.  Std.Err.   z    P>|z| [0.025 0.975]
------------------------------------------------------------------------
Intercept                     48.549    3.685 13.174 0.000 41.326 55.772
Group[T.Outgroup]             -1.861    2.763 -0.674 0.501 -7.276  3.554
Generation                    -2.237    0.330 -6.773 0.000 -2.884 -1.589
Group[T.Outgroup]:Generation   0.712    0.467  1.524 0.128 -0.204  1.627
Group Var                    175.745    4.430
========================================================================
```

NEUTRAL 6: MYSTICAL

```
Mixed Linear Model Regression Results
=======================================================================
Model:                  MixedLM      Dependent Variable:      Average
No. Observations:       396          Method:                  REML
No. Groups:             18           Scale:                   199.3345
Min. group size:        22           Log-Likelihood:          -1622.1947
Max. group size:        22           Converged:               Yes
Mean group size:        22.0
-----------------------------------------------------------------------
                             Coef.  Std.Err.   z    P>|z| [0.025 0.975]
-----------------------------------------------------------------------
Intercept                    50.875    2.461 20.673 0.000 46.051 55.698
Group[T.Outgroup]             2.611    2.655  0.984 0.325 -2.592  7.814
Generation                   -0.372    0.317 -1.174 0.241 -0.994  0.250
Group[T.Outgroup]:Generation -1.818    0.449 -4.050 0.000 -2.697 -0.938
Group Var                    45.590    1.357
=======================================================================
```

ADDITIONAL 1: ATTRACTIVE

```
Mixed Linear Model Regression Results
=======================================================================
Model:                  MixedLM      Dependent Variable:      Average
No. Observations:       396          Method:                  REML
No. Groups:             18           Scale:                   154.0752
Min. group size:        22           Log-Likelihood:          -1574.6290
Max. group size:        22           Converged:               Yes
Mean group size:        22.0
-----------------------------------------------------------------------
                             Coef.  Std.Err.   z    P>|z| [0.025 0.975]
-----------------------------------------------------------------------
Intercept                    51.202    2.375 21.559 0.000 46.547 55.857
Group[T.Outgroup]             2.853    2.334  1.222 0.222 -1.722  7.427
Generation                    0.180    0.279  0.646 0.518 -0.366  0.727
Group[T.Outgroup]:Generation -2.002    0.395 -5.075 0.000 -2.775 -1.229
```

```
Group Var                       52.505    1.681
==========================================================================
```

ADDITIONAL 2: POLITICAL

```
Mixed Linear Model Regression Results
===========================================================================
Model:                  MixedLM      Dependent Variable:       Average
No. Observations:       396          Method:                   REML
No. Groups:             18           Scale:                    164.9657
Min. group size:        22           Log-Likelihood:           -1599.3181
Max. group size:        22           Converged:                Yes
Mean group size:        22.0
---------------------------------------------------------------------------
                              Coef.  Std.Err.   z    P>|z| [0.025 0.975]
---------------------------------------------------------------------------
Intercept                     46.701    3.985 11.719 0.000 38.890 54.511
Group[T.Outgroup]             -1.356    2.415 -0.562 0.574 -6.089  3.377
Generation                    -1.765    0.289 -6.115 0.000 -2.331 -1.199
Group[T.Outgroup]:Generation   0.388    0.408  0.952 0.341 -0.412  1.189
Group Var                    233.350    6.575
===========================================================================
```

ADDITIONAL 3: RELIGIOUS

```
Mixed Linear Model Regression Results
==========================================================================
Model:                 MixedLM      Dependent Variable:       Average
No. Observations:      396          Method:                   REML
No. Groups:            18           Scale:                    252.3893
Min. group size:       22           Log-Likelihood:           -1664.2000
Max. group size:       22           Converged:                Yes
Mean group size:       22.0
--------------------------------------------------------------------------
                             Coef.  Std.Err.   z    P>|z| [0.025 0.975]
--------------------------------------------------------------------------
Intercept                    50.536    2.481 20.368 0.000 45.673 55.399
Group[T.Outgroup]            -0.177    2.987 -0.059 0.953 -6.031  5.678
Generation                   -0.798    0.357 -2.236 0.025 -1.498 -0.098
Group[T.Outgroup]:Generation -0.892    0.505 -1.767 0.077 -1.882  0.098
Group Var                    30.506    0.927
==========================================================================
```
